# Unlocking the biotechnological potential of Baltic microorganisms

**DOI:** 10.3389/fmicb.2025.1682611

**Published:** 2025-10-28

**Authors:** Hanna Mazur-Marzec, Łukasz Grabowski, Alicja Węgrzyn, Agata Błaszczyk, Marta Cegłowska, Przemysław Dąbek, Momina Farooq, Ewa Górecka, Agata Jurczak-Kurek, Anna-Karina Kaczorowska, Tadeusz Kaczorowski, Marija Kataržytė, Robert Konkel, Ewa Kotlarska, Donata Overlingė, Waldemar Surosz, Anna Toruńska-Sitarz, Semko Walat, Monika Zielenkiewicz, Grzegorz Węgrzyn

**Affiliations:** ^1^Department of Marine Biology and Biotechnology, University of Gdańsk, Gdynia, Poland; ^2^Department of Molecular Biology, University of Gdańsk, Gdańsk, Poland; ^3^University Center for Applied and Interdisciplinary Research, University of Gdańsk, Gdańsk, Poland; ^4^Department of Marine Chemistry and Biochemistry, Institute of Oceanology, Polish Academy of Sciences, Sopot, Poland; ^5^Institute of Marine and Environmental Sciences, University of Szczecin, Szczecin, Poland; ^6^Department of Evolutionary Genetics and Biosystematics, University of Gdańsk, Gdańsk, Poland; ^7^Collection of Plasmids and Microorganisms, University of Gdańsk, Gdansk, Poland; ^8^Laboratory of Extremophiles Biology, Department of Microbiology, University of Gdańsk, Gdańsk, Poland; ^9^Marine Research Institute, Klaipeda University, Klaipeda, Lithuania; ^10^Department of Genetics and Marine Biotechnology, Institute of Oceanology, Polish Academy of Sciences, Sopot, Poland; ^11^Department of Phycology, University of Gdańsk, Gdynia, Poland

**Keywords:** added value products, bioactivity, microorganisms, cyanobacteria, bacteriophages, microalgae, fungi, blue biotechnology

## Abstract

Marine microorganisms are increasingly recognized as valuable sources of bioactive compounds and enzymes with diverse applications in biotechnology. Despite its relatively low overall biodiversity, the Baltic Sea harbours a variety of diatoms, dinoflagellates, bacteria (including cyanobacteria), fungi, and bacteriophages with notable biotechnological potential. These organisms produce metabolites with promising application in environmental remediation or as components of pharmaceuticals, nutraceuticals, cosmetics and biomaterials. Enzymes produced by Baltic Sea bacteria catalyse reactions of industrial relevance, while bacteriophages may provide novel tools for pathogen control in aquaculture or serve as sources of genes encoding for valuable enzymes. Although advances in high-throughput genomics and metabolomics have accelerated marine biodiscovery, Baltic Sea microorganisms remain largely understudied and underexploited by industry. This review synthesizes current knowledge on the biotechnological potential of the Baltic Sea microorganisms and highlights opportunities to bridge the gap between basic research and commercial application, particularly in the context of international frameworks such as the Nagoya Protocol.

## Graphical abstract

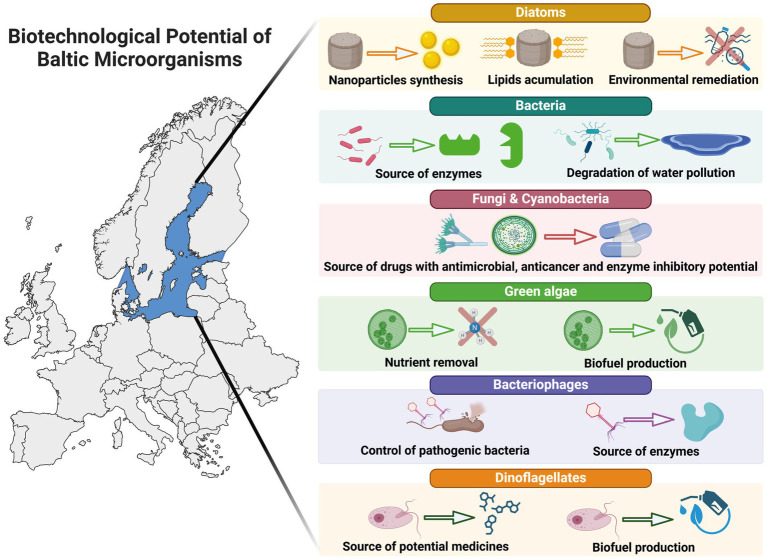

## 1 Introduction

Marine biological resources encompass living organisms, genetic resources, and their products (metabolites), all of which are key components of the marine ecosystem, influencing its diversity, structure, and functioning. Beyond their ecological roles and importance as essential elements of the human diet, people have explored the seas’ natural wealth for various purposes since ancient times. In recent decades, research into the potential of marine organisms as a basis for new, innovative products with significant economic and social implications has grown considerably. This trend is also evident in the Baltic Sea region, where numerous initiatives have been launched to promote the development of marine biotechnology (Supplementary Tables S1–S3). In this area of biotechnology, microorganisms play a particularly pivotal role. The biotechnological potential of marine ecosystems is primarily assessed based on two key factors: the high biological diversity of organisms, which provides a rich and varied source of material for exploration, and the unusual environmental conditions that drive distinctive metabolic processes and increase the likelihood of discovering novel bioactive compounds with unique properties.

The Baltic Sea is commonly regarded as an ecosystem characterised by low biological diversity. However, despite these limitation, the microbial community within this brackish waterbody (excluding viruses) was estimated to encompass over one million (Ojaveer et al., 2010). Microorganisms inhabiting this environment are exposed to pronounced and dynamic gradients in salinity, temperature, and nutrient availability, which impose strong selective pressure. These challenging conditions drive the evolution of specialized physiological adaptation, frequently resulting in the development of unique metabolic pathways and the biosynthesis of structurally and functionally novel biomolecules that are uncommon in microorganisms from more stable ecosystems.

This study aims underscore the underappreciated biotechnological potential of the Baltic Sea microorganisms. Their metabolites and bioactive compounds, developed into low and high added value products, may find applications across a variety of sectors, including pharmaceutical industry, industrial biotechnology or environmental remediation. Importantly, the functional diversity within different microbial taxa suggests that each group may provide different contribution to the development of marine biotechnology sector in the region.

## 2 Low-added value products from the Baltic microorganisms

Value-added products are products that, through processing, acquire additional qualities that contribute to their higher market value compared to the raw materials used to make them. In marine biotechnology, biofuel, food and feed products are defined as low-value, high-volume, and low-risk products (Rotter et al., 2020).

During the last few decades, Baltic Sea microalgae, including diatoms, green algae, cyanobacteria, and dinoflagellates, have gained interest as a potential source of lipids for biofuel production. Most of the research conducted in this area has focused on optimizing microalgae cultivation conditions in order to increase lipid content in cells (Schwenk et al., 2013; Steinhoff et al., 2014; Klin et al., 2020; Klin et al., 2023; Spilling et al., 2021). Nutrient limitation, especially nitrogen, was found to have a beneficial effect on lipid content, but resulted in a decrease in microalgal biomass production (Schwenk et al., 2013; Ratomski and Hawrot-Paw, 2021). Promising results were observed in co-adapted polycultures (microalgae dominated by green algae, protozoans, and bacteria) cultivated under nitrogen limitation and diurnal temperature fluctuations (Mattsson et al., 2019). Under these conditions, the lipid content increased while the biomass remained unaffected. Additionally, the incorporation of elicitors, such as chitosan, into the culture of *Chlorella pyrenoidosa* from the Baltic Sea led to a significant increase in lipid content within a short period (Spilling, 2011). An alternative approach was proposed by Pechsiri and Gröndahl (2022), who suggested that microalgae harvested during extensive and massive blooms of *Nodularia spumigena* in the Baltic Sea could be utilized as a source of biomass for biogas and biofertilizer production.

Due to their ability to uptake nutrients (nitrogen, phosphorus, organic, and inorganic carbon), microalgae have gained significant attention as a component of wastewater treatment processes (Plöhn et al., 2021). In this context, the use of Baltic Sea microalgae (green algae and cyanobacteria) for nutrient removal and biofuel production has been investigated in a couple of studies (Lynch et al., 2015; Jämsä et al., 2017). Wastewater treatment by the green alga *Scenedsmus* sp. UHCC0027 resulted in high nutrient removal rates (100% of ammonium and 90% of phosphate). The high efficiency of this process was achieved when an increased CO_2_ level was applied. However, the regulatory levels for nitrogen and phosphorus removal in treated water were unmet (Lynch et al., 2015). The same strain was used in a subsequent study, where at temperatures between 7 and 13 °C, an effective nutrient removal was recorded with a simultaneous increase in the total lipid content. However, due to the high content of polyunsaturated fatty acids, which are undesirable components of biodiesel, the algal biomass did not meet current standards set by the European Union and the United States (Jämsä et al., 2017).

Furthermore, the Baltic Sea microalge demonstrated effective CO_2_ capture (20–60%) from flue gas (Olofsson et al., 2015; Lindehoff et al., 2024). The results showed both high productivity and the excellent quality of microalgal biomass harvested from these cultures. Additionally, selected strains of Baltic cyanobacteria were found to produce significant amounts of hydrogen gas (H_2_), which is considered a clean energy carrier and a promising alternative to fossil fuels (Khetkorn et al., 2017; Allahverdiyeva et al., 2010).

Few studies have addressed the economic evaluation of the commercial application of Baltic microalgae as a source of low-value products. Pechsiri and Gröndahl (2022) determined the energy return on investment (EROI = total energy output/total primary energy input), as an indicator of environmental and economic performance, for the biomass harvesting process of surface accumulated cyanobacteria from the Gotland basin and its conversion to biogas and biofertilizers. They found that depending on biomass harvesting and transport methods, as well as the type of the obtained products, harvesting toxic *Nodularia spumigena* during blooms from the Baltic Sea could be both environmentally and economically beneficial (Pechsiri and Gröndahl, 2022).

Overall, Baltic Sea microalgae gained academic interest as potential sources of low-value products; however, none have been introduced to the market. The most common challenge lies in scaling up the systems and developing cost-effective outdoor bioreactors (Allahverdiyeva et al., 2010; Steinhoff et al., 2014; Olofsson et al., 2015; Hawrot-Paw and Ratomski, 2024). In particular, the biomass harvesting and dewatering processes remain challenging. Potential solutions for simple and low-cost flocculation and dewatering techniques in closed bioreactor systems using Baltic Sea diatoms and green algae as surrogate species were proposed by Spilling (2011). Future progress will depend on the integration of technological innovations with economic and environmental assessments, paving the way for sustainable large-scale applications within the bioeconomy.

## 3 Baltic Sea diatoms as a source of high-value products

**Figure 1:**
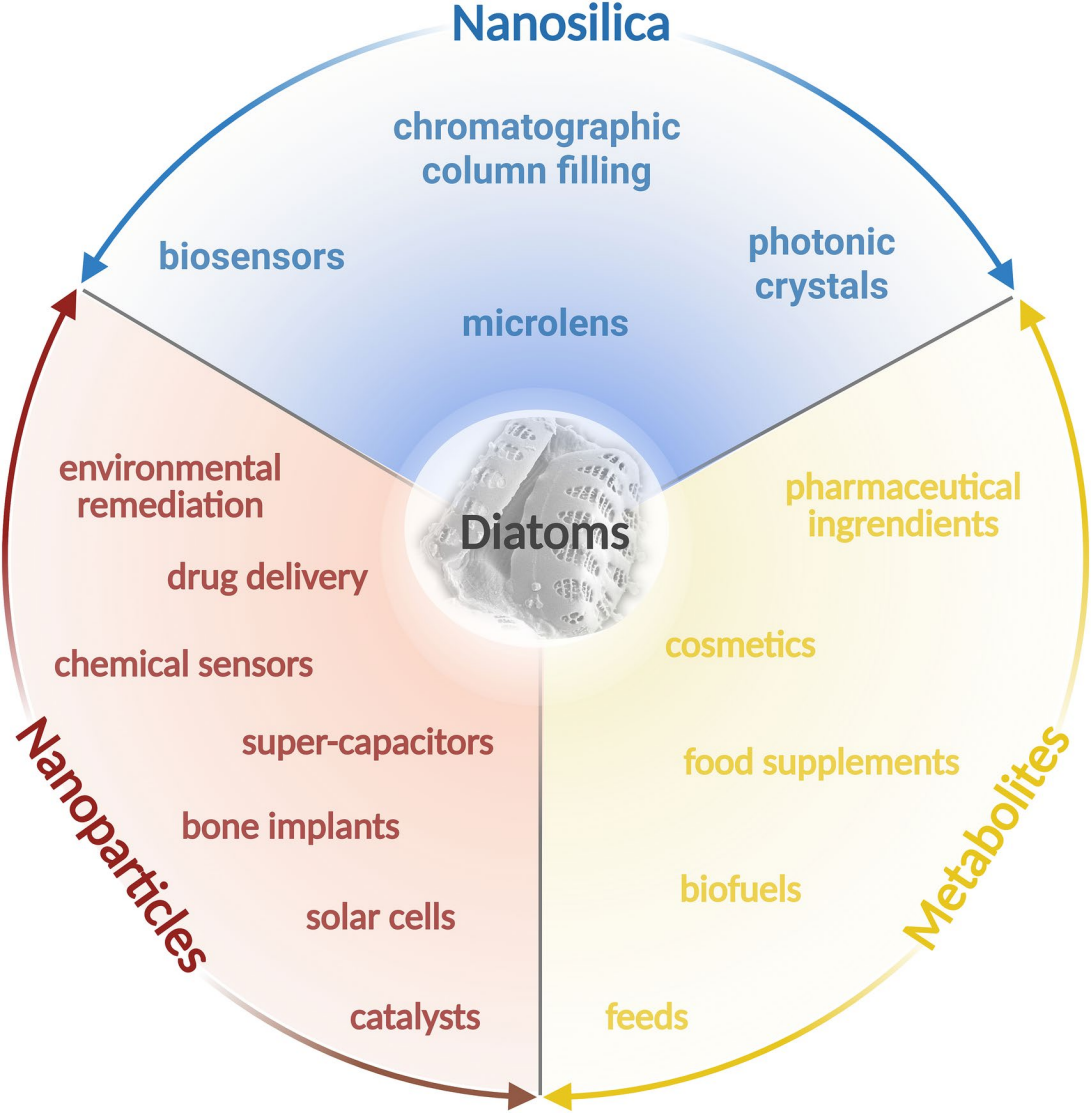
Baltic diatoms and their biotechnological potential. The figure was created using BioRender.com.

Diatoms are an incredibly diverse group of microorganisms which possess significant biotechnological potential (Figure 1). Their exoskeleton, known as frustule, primarily composed of silica (SiO_2_) exhibits intricate ornamentation patterns with diverse shapes and sizes. The silica frustule is durable, transparent, and resistant to degradation (Mishra et al., 2017; Roychoudhury et al., 2022a; Saoud et al., 2022; Zobi, 2022). The frustules, with a large surface area, and high adsorption capacity, represent promising and eco-friendly ‘silica resource’ offering unique properties and significant potential for application in material engineering, nanotechnology, catalysis, and environmental remediation (Golubeva et al., 2023). Moreover, the combination of various metabolic capabilities allows diatoms to uptake, transport, and metabolize metal ions, facilitating the formation of metal nanoparticles both inside and outside their cells. The exact mechanisms of metal nanoparticle synthesis in diatoms are still an area of active research, and the specific enzymes, biomolecules, and cellular processes involved may differ between diatom species and metal ions (Roychoudhury et al., 2022a).

Recently, diatoms from the Baltic Sea have been used in several studies as a source of high-value compounds, including nanosilica, in the fabrication of metal-biosilica complexes or fatty acids. Two species *Gedaniella mutabilis* and *G. flavovirens,* isolated from the Gulf of Gdańsk and Resko Przymorskie Lake, respectively, when treated with a 9 mM silver nitrate solution, were effective in synthesizing Ag-SiO_2_ flower-shaped nanoparticles, with a diameter of 100–500 nm (Roychoudhury et al., 2021). These nanohybrids exhibited autofluorescence properties in the blue light region (excitation ~450–490 nm), and their catalytic activity in the degradation of methylene blue, a dye commonly used in industry, was also demonstrated.

*Pseudostaurosira trainorii* has recently become the Baltic Sea model species in applied research. It has been utilized in the biosynthesis of spindle-shaped iron-oxide nanoparticles (IONPs) (Roychoudhury et al., 2022b). In this work, IONPs diatom bioreagent exhibited catalytic properties in the degradation of nitrophenol after 3 h of reaction. In another study, *P. trainorii* was used to synthesize diatom-based metal nanoparticles (Sprynskyy et al., 2021). The authors conducted a photocatalysis using biosilica doped with palladium(II) chloride nanoparticles to degrade methyl orange in aqueous solution under UV radiation. The degradation efficiency rate reached more than 98% after 75 min. Furthermore, Brzozowska et al. (2022) assessed the optical and physicochemical properties of the latter species by metabolic insertion of titanium ions into the culture medium, achieving a titanium content of more than 8.5% wt. in the diatom frustules. As a result, a reinforced, three-dimensional biosilica enriched with titanium ions was obtained, exhibiting promising semiconductor and optoelectronic properties. The material was characterised by four types of photoluminescence activity in ultraviolet, narrow blue, green, and red-light regions (Brzozowska et al., 2022). The same species was also used in a study by Nowak et al. (2020), where *P. trainorii* biomass served as a source of biosilica and carbon with electrochemical properties. After pyrolyzing the biomass at the temperature of 600 °C, it was found that the carbonized material had a specific discharge capacity of 460 mAh/g^−1^, demonstrating that diatom biosilica can become an electroactive material, potentially valuable for applications such as lithium-ion batteries (Nowak et al., 2020).

Diatoms are also well known for their ability to accumulate lipids within their cells, particularly triacylglycerols (TAGs), as storage compounds (Leyland et al., 2020; Yi et al., 2017). These lipids make diatoms, especially those from epiphytic and benthic communities, a primary food source for micrograzers and higher trophic-level organisms in the world’s most productive coastal areas (Cox et al., 2020). Diatoms can adjust their lipid biosynthesis in response to various environmental stressors, such as nutrient and light limitation (d'Ippolito et al., 2015; Hildebrand et al., 2012; Sayanova et al., 2017; Singha et al., 2022). They are also a storehouse for a wide range of bioactive compounds, including pigments, and polyunsaturated fatty acids (PUFAs), such as omega-3 (Bayu et al., 2020; Nieri et al., 2023). Due to antioxidant, antibacterial, anticancer and other activities, these compounds have potential applications in the pharmaceutical and nutraceutical biotechnological sectors as a starting material for the development of biomaterials, drugs, dietary supplements, and functional foods (Mal et al., 2021; Marzec et al., 2022; Sharma et al., 2021).

With a growing demand for eco-friendly alternative energy sources and nutraceutical components, more research is being conducted on the topic of cold-water microalgae with high lipid yield potential. A detailed study of two diatom species commonly found in the Baltic Sea waters, *Chaetoceros wighamii* Brightwell and *Thalassiosira baltica* (Grunow) Ostenfeld, revealed a significant variation in their fatty acids (FA) profiles depending on temperature, light, and nutrient limitation (Spilling et al., 2021). Both diatoms were confirmed to contain eicosapentaenoic acis (EPA), but the concentration of this PUFA in *T. baltica* was generally high (with a maximum of approximately 30 mg EPA/g of ash-free dry weight under Si-limitation) when compared to other diatom species *C. wighamii.*

The effect of light intensity on EPA production was studied by Spilling et al. (2021). The authors found that the fraction of EPA was lower under high light conditions, but no correlation was observed between overall FA content and light intensity. The most significant factors affecting the FA profile in these diatoms were the growth phase and nutrient limitation pattern. A similar conclusion regarding the relationship between FA profile and growth phase was reached in another study that explored factors influencing lipid content in Baltic diatoms and other brackish and marine microalgae (Schwenk et al., 2013). It was found that FA content increased by an average of 2.6 times as cultures transitioned from the exponential to the stationary phase. The collected data strongly support the hypothesis that the growth phase, regardless of nutrient limitation, is a significant factor affecting the total FA content in diatom cells.

The challenges that the cultivation of diatom for biomass in the Baltic Sea region may face include the variation in seasonal environmental conditions. While outdoor culturing systems are most efficient in areas with a warm, mild climate, temperature and light intensity fluctuations could affect cultivated diatoms’ growth and fatty acid content. This disadvantage is worth considering, as the amount and quality of the yield from diatom cultivation systems are essential for the competitiveness of the final product (Gilmour, 2019). The simulation of seasonal variation in culture conditions by Cheregi et al. (2021) shows that the same species cannot be grown throughout the year; instead, a seasonal species rotation should be applied, using local species that reach maximum growth at temperatures matching the seasonal conditions. An example of the advantages of large-scale diatom cultivation is the ability to combine biomass growth with sustainable methods for removing waste gases. A screening study of the most promising temperate climate diatom species, *T. baltica* and *C. wighamii* (Spilling, 2011), demonstrated the potential for diatom cultivation combined with CO_2_ fixation from industrial exhaust gases, such as those from geothermal power plant fumes. The source of CO_2_ did not affect growth efficiency when compared to pure, commercial CO_2_. However, the potential impact of pollutants, such as SO_2_ and other toxic gases, present in CO_2_ sources on diatom growth remains to be investigated in further research. Such studies may provide further insights into the sustainability and viability of this cultivation method.

In conclusion, Baltic diatoms can represent a significant and sustainable source of high-value products due to their diverse metabolic capabilities, efficient lipid accumulation, and ability to synthesize metal nanoparticles. With ongoing research into optimizing cultivation conditions and extraction methods, diatoms could play a pivotal role in advancing biotechnological applications in materials science, environmental remediation, and the pharmaceutical sector.

## 4 Dinoflagellates phycotoxins as potential pharmaceuticals

**Figure 2:**
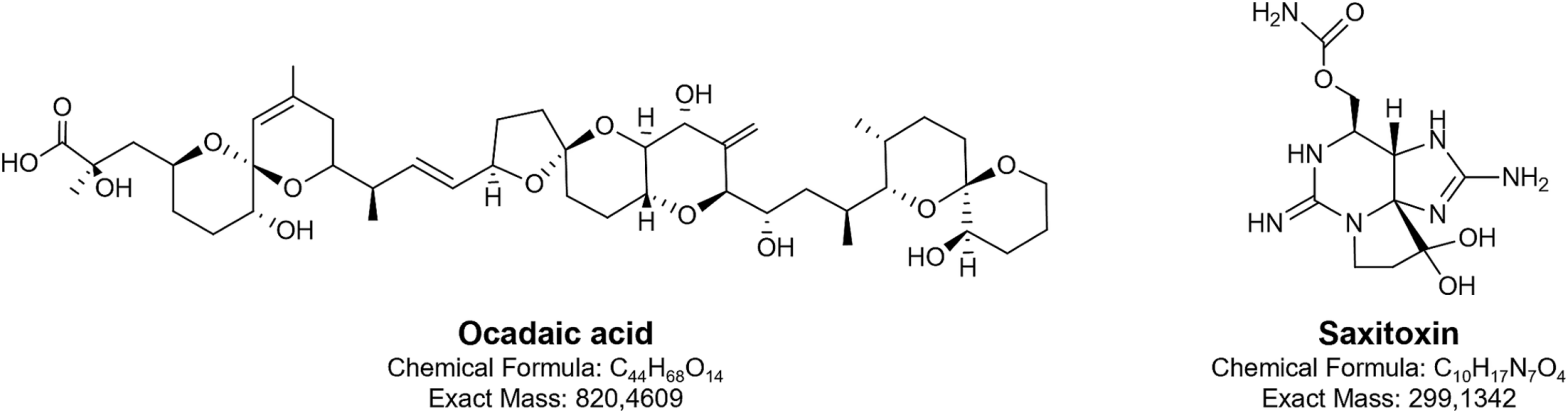
Chemical structures of two toxins produced by Baltic dinoflagellates: okadaic acid and saxitoxin.

Phycotoxins produced by dinoflagellates cause significant damage to the global fishing industry and aquaculture. These toxins are also responsible for various human diseases (Karlson et al., 2021). Despite their harmful effects, some of these metabolites have been investigated as potential candidates for developing high-value products (Cho et al., 2020). The Baltic population of *Alexandrium ostenfeldii* produces saxitoxins (STXs), the alkaloids with guanidine group (Figure 2), responsible for paralytic shellfish poisoning (PSP) (Salgado et al., 2015).

Over 60 structural variants of saxitoxins have been identified, though in *Alexandrium ostenfeldii* from the Baltic region, only saxitoxin (STX) with a carbamoyl group and two decarbamoyl gonyautoxins, GTX-3 and GTX-2, have been detected. Among these, the neurotoxic activity of the carbamoyl analogues is the most potent. STXs selectively bind to voltage-gated sodium channels (Na_v_s), particularly at site 1 on the *α*-subunit, and physically block the pore of the channels. This prevents sodium ion influx during membrane depolarization, thereby inhibiting the initiation and propagation of action potentials in excitable cells, such as neurons and muscle fibres (Wiese et al., 2010; Stevens et al., 2011; Shen et al., 2018). The blockade of Na_v_-mediated sodium currents leads to the interruption of neuronal signalling and neuromuscular transmission, which ultimately results in paralysis. Owing to this specific interaction with Na_v_ channels, STXs have been explored for potential applications as local anaesthetics or anti-wrinkle agents, where controlled inhibition of nerve conduction is desirable (Kohane et al., 2000; Epstein-Barash et al., 2009). The drug-like properties of saxitoxins, particularly the decarbamoyl analogues, have been confirmed by Flores-Holguín et al. (2024) using the SwissADME web tool. In the 1950s and 1960s, due to its acute toxicity, saxitoxin was also investigated as a potential chemical weapon (Sierra and Martínez-Álvarez, 2020).

Okadaic acid (OA) and dinophysiotoxin DTX-1 (methylated form of OA) produced in the Baltic by *Dynophysis* species are polyether fatty acids (Figure 2) and represent the most widely occurring toxins associated with diarrhetic shellfish poisoning (DSP). OA and DTX-1 exert their biological activity primarily through potent inhibition of serine/threonine-specific protein phosphatases PP1 and PP2A by binding to their catalytic subunits. This inhibition disrupts the balance between phosphorylation and dephosphorylation in cells, leading to abnormal hyperphosphorylation of numerous intracellular proteins. As a result, the function of many signalling and regulatory pathways becomes impaired (Valdiglesias et al., 2013; Lee et al., 2016). The downstream effects of this molecular disruption include gastrointestinal symptoms such as diarrhoea, vomiting, and abdominal pain. Furthermore, OA has been shown to induce both cytotoxic (Valdiglesias et al., 2011) and neurotoxic effects (Kamat et al., 2013). Because of its precise and potent inhibition of PP1 and PP2A, OA is widely used as a research tool to study intracellular signalling pathways, particularly those involving reversible phosphorylation. It has been instrumental in elucidating the role of phosphatases in processes such as cell cycle regulation, apoptosis, and neurodegeneration, including mechanisms implicated in Alzheimer’s disease (Cruz et al., 2013; Kamat et al., 2013). Furthermore, the immunoregulatory potential of OA has been demonstrated (López et al., 2011). In experiments involving the mouse T lymphocyte cell line EL-4, exposure to OA led to down regulation of the T cell receptor complex. A similar effect was observed with yessotoxin, another polyether toxin produced by dinoflagellates, although its mechanism of action differed (López et al., 2011).

Biotechnological applications of dinoflagellate metabolites, including toxins associated with PSP and DSP, have been extensively reviewed in several studies (Camacho et al., 2007; Assunção et al., 2017; Cho et al., 2020). A compilation of patents highlights the diverse therapeutic potential of the toxins (Camacho et al., 2007; Assunção et al., 2017). However, it has been noted that the limited quantities of available metabolites, in relation to the amounts required for preclinical studies, pose a significant challenge to advancing research on their therapeutic potential. The production of these metabolites in bioreactors, is insufficient for commercialization, primarily due to the slow growth rate of dinoflagellates. Furthermore, genetic modifications to enhance the efficiency of production processes are more complex than in microorganisms with smaller genomes. The number of sequenced dinoflagellate genomes is also notably limited in comparison to other microbial species (Lin, 2024). Moreover, chemical synthesis of these compounds is challenging, involving numerous steps, which significantly impact both the cost and availability of the product. Addressing these challenges will likely pave the way for developing at least some of dinoflagellate metabolites into viable therapeutic agents.

## 5 Baltic cyanobacteria a treasure trove of bioactive compounds

Summer cyanobacteria blooms are among the most problematic issues of the Baltic Sea. They generate high biomass and disrupt the functioning of the ecosystem (Karjalainen et al., 2007). Regardless of their negative environmental impact, cyanobacteria are a valuable source of compounds that can serve as a basis for developing high-value products (Luesch et al., 2025; Baunach et al., 2024; Demay et al., 2019). A particularly prominent example in this context is dolastatin, which has served as a lead compound for the development of several anticancer agents (Luesch et al., 2025). However, as its 40-year development pathway illustrates, the process of translating a biologically active compound into a marketable pharmaceutical remains both time-consuming and resource-intensive. Thus far, the biotechnological and pharmaceutical potential of Baltic cyanobacteria has been explored using filamentous forms from the order *Nostocales*, such as *Nodularia*, *Anabaena*, and *Nostoc* (Fewer et al., 2009; Jokela et al., 2012; Mazur-Marzec et al., 2018; Fewer et al., 2021; Konkel et al., 2023a,b). These microorganisms produce secondary metabolites with antifungal, antiviral, anticancer, and enzyme-inhibiting activities.

**Figure 3:**
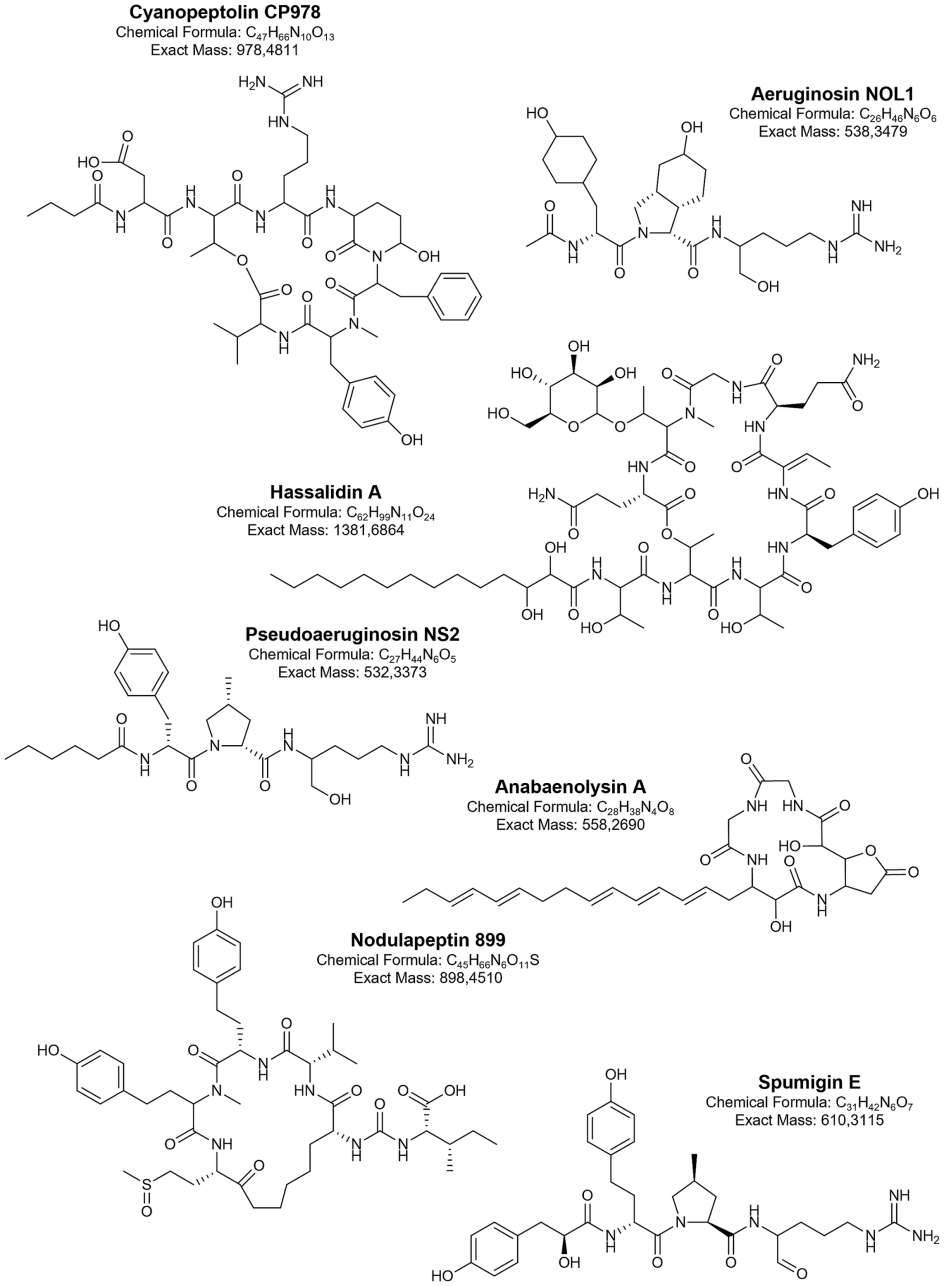
Biologically active metabolites produced by Baltic cyanobacteria.

Antifungal cyanometabolites such as hassallidins, hassallidin-like balticidins, puwainaphycins, and anabaenolysins are cyclic lipopeptides (Fewer et al., 2021). They contain a peptide ring and a fatty acid chain, and each of these structural elements has specific characteristics for a given class of lipopeptides (Figure 3). Hassallidins have been identified in various filamentous cyanobacteria, including the Baltic strains of *Anabaena* (Bui et al., 2014; Vestola et al., 2014). These compounds contain a peptide ring with eight amino acids linked to a saturated fatty acid chain (C14-18) through the exocyclic residue (Bui et al., 2014; Vestola et al., 2014). In addition to chlorine (in balticidins), different types and numbers of monosaccharides have been identified in the structure of hassallidins. Puwainaphycins, another class of cyclic lipopeptides produced by cyanobacteria (Vašíček et al., 2020), were detected in the benthic *Nodularia harveyana* strain UHCC-0300 (Saurav et al., 2022). Puwainaphycins (and structurally homologous minutissamides) are composed of nine amino acids and a *β*-amino-*α*-hydroxy-*γ*-methyl fatty acid incorporated into the ring (Gregson et al., 1992; Vašíček et al., 2020; Saurav et al., 2022). The unbranched fatty acid part (C10-18) of the molecule can be substituted by chlorine, hydroxyl, or oxo functional groups. Anabaenolysins were discovered in *Anabaena* strains from the Gulf of Finland (Jokela et al., 2012; Oftedal et al., 2012). In the structure of these lipopeptides, four amino acids, including the unusual 2-(3-amino-5-oxytetrahydrofuran-2-yl)-2-hydroxyacetic acid, and fatty acid chain (C18) with different degrees of unsaturation are present (Jokela et al., 2012; Oftedal et al., 2012).

All these lipopeptides exhibit activity against human pathogenic fungi (Neuhof et al., 2005; Bui et al., 2014; Vestola et al., 2014; Shishido et al., 2015; Humisto et al., 2019; Hájek et al., 2021). They act by disintegrating sterol-containing cell surface membranes (Jokela et al., 2012; Oftedal et al., 2012; Hrouzek et al., 2012; Humisto et al., 2019). The effects clearly depend on the structure of the lipophilic part of the molecules, particularly the length of the fatty acid chain and the type and position of the substituted functional group (Hájek et al., 2021). For example, puwainaphycins with longer fatty acid chains exhibit more potent antifungal properties compared to compounds with shorter chains, while hydroxylation of part of the lipopeptides significantly reduces their activity (Hrouzek et al., 2012; Hájek et al., 2021; Saurav et al., 2022). In addition to antifungal activity, cyanobacterial lipopeptides also cause lysis, necrotic changes, and death of various eukaryotic cells through interaction with sterol-containing membranes (Hrouzek et al., 2012; Jokela et al., 2012; Oftedal et al., 2012; Humisto et al., 2019). Since the cytotoxicity of cyanobacterial lipopeptides has also been observed in healthy cells, their biotechnological application would require structural modifications to eliminate the undesirable effects while preserving antifungal activity.

Serine protease inhibitors are another important class of bioactive metabolites detected in Baltic cyanobacteria. Serine proteases are involved in many essential metabolic pathways and are found in both prokaryotic and eukaryotic cells. Disruption of their balanced activity can lead to various pathologies, including cancer, inflammation, cardiovascular disease, and neurodegenerative disorders (Patel, 2017; Harish and Uppuluri, 2018; Singh et al., 2023). All of this makes serine proteases important targets in the drug discovery process.

The activity against serine proteases is demonstrated by several classes of linear and cyclic peptides produced by Baltic cyanobacteria. These include non-ribosomally synthesised spumigins, aeruginosins, pseudoaeruginosins, anabaenopeptins, cyanopeptolins, and nostocyclopeptides. Different structural variants of linear spumigins, aeruginosins, and pseudoaeruginosins have been identified in many Baltic strains of *N. spumigena* (Fewer et al., 2009; Mazur-Marzec et al., 2012; Liu et al., 2015; Mazur-Marzec et al., 2016), *Nostoc* spp. from the Gulf of Finland (Heinilä et al., 2022), and *Aphanizomenon* sp. strain KUCC C2 from the Curonian Lagoon (Overlingė et al., 2024a). All these tetrapeptides contain arginine (Arg) or its mimetics at the *C*-terminal position. The presence and form of Arg have a significant impact on the biological activity of these peptides (Liu et al., 2023). At the *N*-terminal part of aeruginosins and pseudoaeruginosins, a short fatty acid chain is present. In spumigins and varlaxins, this position is occupied by hydroxyphenyl lactic acid (Hpla) or 2-(4-hydroxyphenyl) acetic acid (Hpaa), respectively. In spumigins and pseudoaeruginosins, the position adjacent to Arg contains proline or methylproline (MePro), while in aeruginosins and varlaxins, a 2-carboxy-6-hydroxyoctahydroindole (Choi) is present (Figure 3).

Although these peptides are common components of Baltic cyanobacterial cells, only a few have been isolated and tested in enzyme inhibition assays. Spumigin E (Fewer et al., 2009), varlaxins (1046A and 1022A) (Heinilä et al., 2022), and pseudoaeruginosin NL2 (Liu et al., 2015) inhibited porcine or/and human trypsins with IC_50_ in the nanomolar range, while aeruginosin AER525 was most active against thrombin (IC_50_ = 0.59 μM), it also inhibited trypsin and carboxypeptidase A (Overlingė et al., 2024b). A comparison of the biological activity of peptides from the described classes (Liu et al., 2023) indicates that those produced by Baltic strains are among the strongest inhibitors of trypsin and thrombin. The significance of serine protease inhibitors is emphasized by the growing body of research on their potential use in cancer treatment. For example, trypsin-3 inhibitors have been shown to reduce metastasis in prostate, breast, and ovarian cancers (Sananes et al., 2023). The link between trypsin inhibition and anticancer activity has also been observed for suomilide from the Baltic *N. sphaerocarpa* UHCC 0038 (Ahmed et al., 2021). This compound, which shares some structural similarities with aeruginosins (Figure 3), was previously identified in *N. spumigena* strains AV1 and HKVV (Fujii et al., 1997). Suomilide isolated from *N. sphaerocarpa* UHCC 0038 inhibited the activity of recombinant human trypsins (1, 2, and 3) at nanomolar concentrations (4.7–104.0 nM) and, at 3.3 μM, also inhibited the invasion of aggressive metastatic PC-3 M prostate cancer cells (trypsin-3) (Ahmed et al., 2021).

Cyanopeptolins are another class of serine protease inhibitors commonly found in cyanobacteria from various taxonomic groups (Weckesser et al., 1996; Rounge et al., 2007; Jones et al., 2021). In the Baltic Sea, their presence has been detected in *Nostoc edaphicum* CCNP1411 (93 structural variants) (Mazur-Marzec et al., 2018; Konkel et al., 2023a) and in bloom samples from the Curonian Lagoon (Overlingė et al., 2021). The unique feature of cyanopeptolins is the presence of 3-amino-6-hydroxy-2-piperidone (Ahp) in a six-amino acid peptide ring cyclized by an ester bond (Figure 3). They also have a side chain composed of 1–2 amino acids and a short fatty acid (Weckesser et al., 1996; Konkel et al., 2023a). Cyanopeptolins isolated from the Baltic *N. edaphicum* CCNP1411 inhibited the activity of trypsin, chymotrypsin, and elastase in the low μM range. The activity against these enzymes was selective and depended on the type of amino acid in the second position, between conserved threonine and Ahp (Figure 3). Although all of these depsipeptides were active against chymotrypsin, those containing Arg in this position also inhibited trypsin, while elastase was only inhibited by cyanopeptolins with leucine (Konkel et al., 2023a). Interestingly, two of the leucine-containing cyanopeptolins and CP978 (with Arg) also showed cytotoxic effects against human cervical cancer cells (HeLa). Cyanopeptolin CP978 appears to be one of the most interesting peptides produced by Baltic cyanobacteria in terms of potential applications. In addition to inhibiting the activity of serine proteases, it also protects human cells from SARS-CoV-2 infection (Konkel et al., 2023b). CP978 interacts with the virion by binding directly to the coronaviral S protein. This action caused a significant decline in SARS-CoV-2 replication in A549^ACE2/^™^PRSS2^ cells (adenocarcinoma human alveolar basal epithelial cells overexpressing ACE2 and TMPRSS2) and in primary human airway epithelial cells (HAE) (Konkel et al., 2023b). In the viral infection assay, the determined IC_50_ was 82 nM, classifying CP978 as one of the most potent natural products inhibiting SARS-CoV-2 infection (Wang et al., 2022). The antiviral activities of other cyanometabolites (e.g., lectins, sulphated polysaccharides) have also been described, but their presence in Baltic cyanobacteria has never been explored.

Similarly to cyanobacteria from other aquatic environments (Monteiro et al., 2021; Zervou et al., 2021), the Baltic strains produce numerous variants of cyclic hexapeptides classified as anabaenopeptins. These peptides have been identified in *N. spumigena* strains (Rouhiainen et al., 2010; Mazur-Marzec et al., 2012; Mazur-Marzec et al., 2016), *N. edaphicum* CCNP1411 (Konkel et al., 2022), picocyanobacterium *Coelosphaeriaceae* 06S067 (Häggqvist et al., 2016), *Aphanizomenon* sp. KUCC C1 (Overlingė et al., 2024a), and in bloom samples dominated by *N. spumigena*, *Aphanizomenon* sp., and *Dolichospermum* sp. (Spoof et al., 2015; Overlingė et al., 2021). A characteristic feature of this class of compounds is the cyclic pentapeptide ring, with a conserved D-lysine linked to the exocyclic amino acid through the ureido bond (Figure 3). Anabaenopeptins identified in Baltic cyanobacteria inhibit protein phosphatase and carboxypeptidase A (IC_50_ in the nanomolar range) (Spoof et al., 2015; Häggqvist et al., 2016); their activity against elastase has also been documented (Konkel et al., 2022). The potency of the effects on tested enzymes depends mainly on the type of amino acid in the exocyclic position. The pharmaceutical potential of anabaenopeptins (from *Planktothrix* and *Nostoc*) with Arg in this position has been well documented by Schreuder et al. (2016). These peptides, as strong inhibitors of thrombin-activable fibrinolysis (TAFIa) (IC_50_ = 1.5–16 nM), could be important agents in the treatment and prevention of thrombosis. Although structural variants of anabaenopeptins with Arg in the exocyclic position are produced by Baltic cyanobacteria (Häggqvist et al., 2016; Overlingė et al., 2021; Overlingė et al., 2024a), their activity against TAFIa has never been tested.

In addition to the extensive body of research on isolated and identified cyanometabolites, bioassays were performed on extracts and fractions from Baltic cyanobacterial (Mazur-Marzec et al., 2015; Felczykowska et al., 2015; Szubert et al., 2018; Cegłowska et al., 2022; Overlingė et al., 2024a). The results of these studies demonstrate that many biologically active metabolites produced by Baltic cyanobacteria remain to be discovered. Notably, several already known cyanometabolites described here exhibit strong antifungal, antiviral, anticancer, and enzyme-inhibitory activities. However, none of them has progressed beyond the stage of basic research. One of the limitations appears to be the insufficient yield of these metabolites from biomass, which hinders efforts to conduct more advanced studies.

## 6 Biotechnological potential of other Baltic bacteria

**Figure 4:**
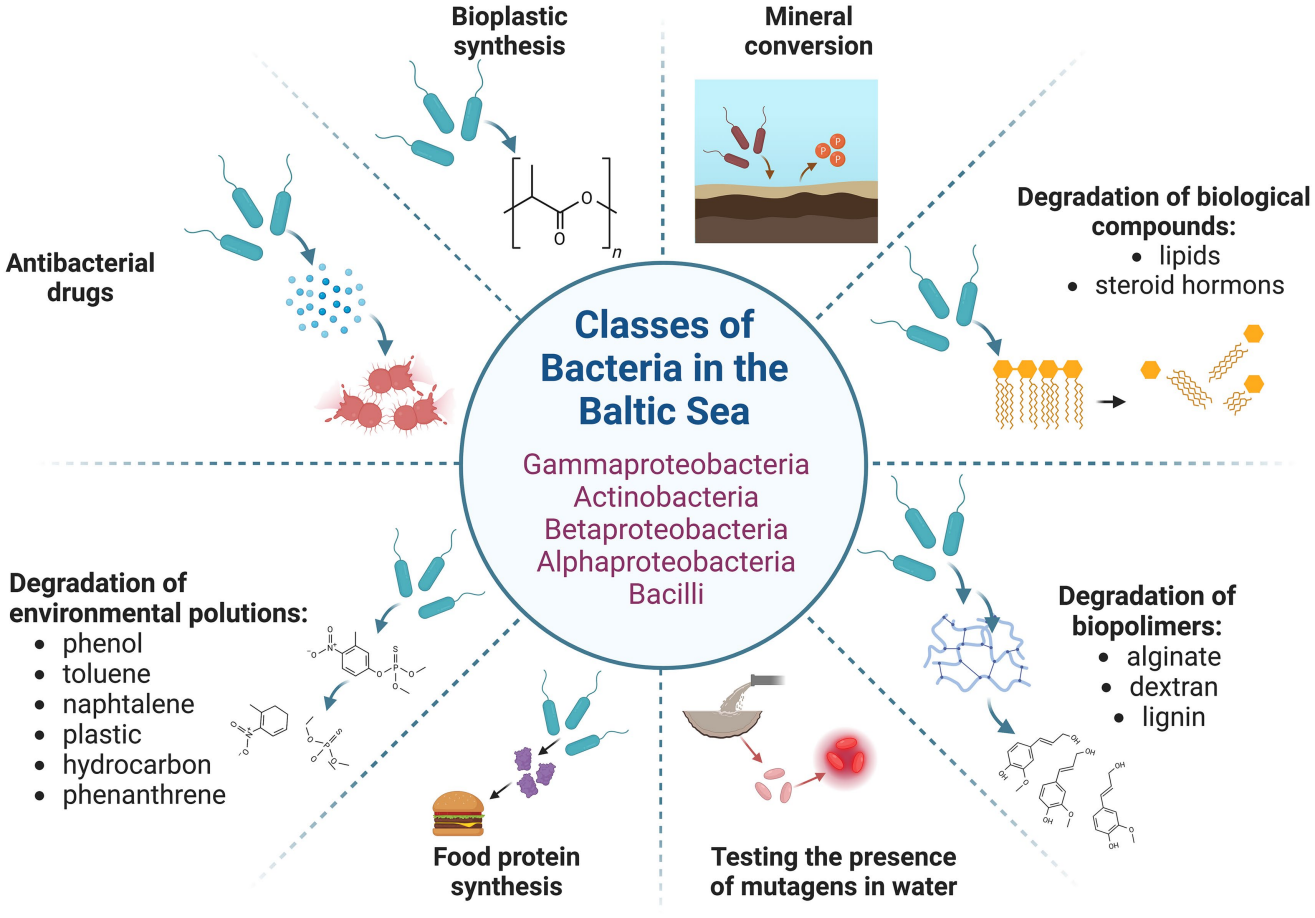
Baltic bacteria and their potential biotechnological application. The figure was created using BioRender.com.

In addition to cyanobacteria, other bacterial species possess significant biotechnological potential due to their diverse capabilities, which can be applied across various sectors of human activity. These microorganisms exhibit a range of functions, from the degradation of uncommon organic compounds and the detection of mutagenic substances to the production of potentially valuable enzymes, antimicrobial agents, and the synthesis of nutrients using H_2_O and CO_2_ as substrates (Ferrer et al., 2019; Barzkar et al., 2024). Bacteria exhibiting such characteristics are also found in the Baltic Sea (Figure 4). Among them, Actinomycetes have emerged as a predominant group of marine bacteria with significant biotechnological relevance (Jagannathan et al., 2021; Ngamcharungchit et al., 2023). The general role of marine bacteria in advancing biotechnological innovations has been reviewed recently in several papers (Ferrer et al., 2019; Jagannathan et al., 2021; Ngamcharungchit et al., 2023; Barzkar et al., 2024). Thus, in this chapter, we will specifically focus on the biotechnological potential of Baltic bacteria (excluding cyanobacteria, which were addressed in the preceding chapter).

### 6.1 Enzymes from Baltic bacteria

When evaluating the biotechnological potential of microorganisms, one of the most apparent applications is the isolation and use of various enzymes that catalyse reactions of technological significance. Indeed, marine bacteria may encode enzymes of atypical activities or characteristics other than those found in species from more extensively explored environments, such as those from the *Entrobacteriaceae* family. These enzymes might be of interest due to the unique environmental conditions under which they function.

Early research led to the cloning and characterisation of a gene encoding a unique hemolysin from *Vibrio pommerensis* from the Baltic Sea (Jores et al., 2003). Hemolysins are of pharmaceutical interest, particularly as potential anticancer enzymes, and may also be utilized in developing biosensors (Panchal et al., 2002). Another group of biotechnologically relevant enzymes are lipases. A gene encoding a lipase active at temperatures as low as 5–10 °C has been cloned with the gene identified in a metagenomic library of Baltic Sea sediment bacteria. Thus, the specific bacterium containing this gene remains unidentified (Hårdeman and Sjöling, 2007). Interestingly, microalgae residing in the Baltic Sea are known to contain relatively high levels of lipids (Dolganyuk et al., 2022), suggesting that bacteria in the same habitat may have evolved to utilize these compounds by producing effective lipases. Another metagenomic analysis revealed that bacteria living in Baltic waters harbour numerous genes coding for transposases (Vigil-Stenman et al., 2017), enzymes responsible for transposition of mobile genetic elements in cells, which hold significant biotechnological potential in genetic engineering.

The chemical degradation of complex organic compounds can be challenging, yet these molecules could serve as valuable sources of molecular monomers. Examples include alginate and dextran, which can be hydrolysed into oligo- or monosaccharides through enzymatic processes. Marine bacteria represent a promising source of alginate lyases (Barzkar et al., 2022a) and dextranases (Barzkar et al., 2022b). Bacterial species that produce these enzymes have been found in the Baltic Sea (Barzkar et al., 2022a,b). Another example are fatty acids and sialic acid degrading enzymes. In this context, a cold-active bacterium isolated from Baltic Sea ice, *Shewanella glacialimarina*, with an interesting fatty acid profile and the ability to metabolize sialic acid (Qasim et al., 2021), could represent a promising step toward the biotechnological application of enzymes derived from this organism.

### 6.2 Potential direct use of bacteria to conduct specific degradation processes

Although enzymes encoded by bacteria can be highly effective under laboratory conditions or as components of pharmaceuticals, the degradation of specific compounds in the environment is typically more efficient when living bacteria are employed rather than isolated enzymes.

Numerous studies have described examples of bacteria capable of degrading a wide range of compounds, including xenobiotics. These microorganisms could assist in the remediation of chemical pollution in the environment. For example, *Pseudomonas* spp. and *Exiguobacterium* sp., isolated from a phenanthrene-enriched, Baltic Sea sediments, revealed the ability to degrade the pollutant (Edlund and Jansson, 2008). Specifically, phenanthrene concentration was decreased from 3.7 μg/mL to 0.8 μg/mL in such sediments, during the incubation period of 60 days, due to the action of these bacteria (Edlund and Jansson, 2008). Isolations of bacteria degrading phenol and toluene from the Baltic Sea have also been reported; the degradation capabilities were assumed on the basis of the presence of genes encoding specific enzymes, phenol hydroxylases and catechol 2,3-dioxygenases (Vedler et al., 2013) and toluene monooxygenase and toluene/xylene monooxygenase (Heinaru et al., 2016). These strains belong to the *Limnobacter* genus and *Pseudomonas stutzeri* species, respectively, and were proposed as potential biocontrol agents for the remediation of phenol and toluene contamination in marine waters. In some cases, the degradation of specific compounds may require the involvement of bacterial communities rather than single species. Such a phenomenon was observed in the degradation of nonylphenol and butyltin by bacteria present in bottom sediments from the Baltic Sea, particularly those from the Gulf of Finland (Kuzikova et al., 2022). Namely, the sediments were enriched with nonylphenol and/or butyltin, up to 300 mg/kg and 95 mg/kg, respectively, and upon incubation for 240 days with bacterial communities consisting with species belonging mainly to genera *Halothiobacillus*, *Geothrix*, *Methanosarcina*, *Dyella*, *Parvibaculum*, *Pseudomonas*, and *Proteiniclasticum*, and order *Rhizobiales*, a total removal of monobutyltin was observed, with the nonylphenol residual rate estimated for 40% (Kuzikova et al., 2022). In fact, the Gulf of Finland seems to be particularly rich in xenobiotic-degrading bacteria, as demonstrated by the isolation of the *Delftia tsuruhatensis* strain, which is effective in degrading naphthalene, as demonstrated by the observation of the 43% degree of naphthalene degradation during about 24 h incubation of this bacterium in a mineral medium containing naphthalene as the only carbon source (Sazonova et al., 2023).

Bacteria that oxidize petroleum-derived pollutants from accidents related to the transportation or processing of petroleum may either exist as free-living organisms in marine waters and sediments or colonize the digestive tracts of fish, as demonstrated in recent studies on samples from the Baltic Sea (Polyak et al., 2020). Majority of the petroleum hydrocarbon-degrading bacteria from this sea belong to *Actinobacteria* and *Betaproteobactera* (Reunamo et al., 2013). It is essential to highlight that the structure of microbial communities can change significantly in the presence of crude oil, shale oil or diesel fuel, as demonstrated by studies on the coastal waters of the Baltic Sea (Viggor et al., 2013). Genomic studies, indicate that Baltic bacteria possess considerable potential for oil degradation, as evidenced by the abundance of *alkB* genes encoding alkane hydroxylases (Viggor et al., 2015). This conclusion was further supported by more comprehensive metagenomic analyses (Miettinen et al., 2019). Notably, recent studies on microbial genomes derived from Baltic Sea samples have shown that the metabolic potential of marine bacteria is closely linked to both the size of their genome and abiotic environmental factors (Rodríguez-Gijón et al., 2023).

Another group of pollutants includes pharmaceuticals, which can enter marine waters through their use and subsequent waste disposal (Wagil et al., 2015; Lindim et al., 2017). Among these, there are hormones, especially those belonging to the steroid group. Baltic bacteria capable of degrading steroid compounds were isolated, offering potential biotechnological applications for removing this kind of contaminants from natural marine environments. Examples of such steroid-degrading bacteria include strains from the *Buttiauxella* genus, which were demonstrated to be able to utilize cholesterol, oestradiol and testosterone as a carbon source when growing in a minimal medium (Zhang et al., 2011). In the case of *Vibrio,* genes encoding enzymes responsible for steroid degradation have been identified (Sang et al., 2012).

### 6.3 Putative drugs produced by Baltic bacteria

Various Baltic bacterial strains produce bioactive compounds with potential pharmaceutical significance. One such example is the *Streptomyces champavatii* strain, which is capable of producing a cyclic octapeptide called champacyclin, known for its antibacterial properties (Pesic et al., 2013).

The synthesis of various antimicrobial compounds has been observed in several Baltic bacterial strains associated with eukaryotic organisms. The proposed biological significance of this symbiosis lies in the protection of the eukaryotic host against other microorganisms, while the host, in turn, provides a favourable microenvironment for the bacterial symbionts. Notable examples of antimicrobial compound-producing Baltic bacteria associated with higher organisms include strains living in close proximity to bryozoan species, *Marinomonas*, *Shewanella*, and *Vibrio* (Heindl et al., 2010; Heindl et al., 2012). Additionally, bacteria associated with the brown alga *Laminaria saccharina* represent multiple species within the *Alphaproteobacteria*, *Betaproteobacteria*, *Gammaproteobacteria*, *Bacteroidetes*, *Firmicutes*, and *Actinobacteria* phyla (Wiese et al., 2009). Further examples include bacteria associated with the soft coral *Alcyonium digitatum*, classified as *Bacillus methylotrophicus* and *Bacillus amyloliquefaciens* (Pham et al., 2016), as well as *Actinobacteria* associated with marine sponges (Schneemann et al., 2010; Kunz et al., 2014). Among these groups, *Actinobacteria* appear to be the most abundant bacterial group in the Baltic Sea, comprising approximately 22–27% of bacterial communities in this environment (Holmfeldt et al., 2009; Jiang et al., 2011). These bacteria are known to produce a wide range of bioactive molecules of significant biotechnological relevance.

### 6.4 Baltic bacteria as metabolizers of minerals, (bio)plastic and lignin, and producers of potential nutrient proteins

Research has shown that bacteria isolated from Baltic bottom sediment contribute to both the corrosion and decomposition of submerged objects and structures, including shipwrecks, sunken objects, underwater pipelines. According to Cybulska et al. (2020), sulfate-reducing bacteria are the most abundant microorganisms involved in these processes and play a key role in microbial corrosion. In contrast, iron-converting bacteria have been found to be considerably less abundant. Moreover, sulfate-reducing bacteria, such as members of the family *Desulfomicobiaceae* identified in the Baltic Sea, have recently been proposed as potential agents responsible for the release of phosphorus from sediments (Cakmak et al., 2024). This is of particular biotechnological interest, as phosphorus is a critical element in eutrophication processes that enhance biological production.

Plastic is one of the most prevalent environmental contaminants, found in virtually all ecosystems, including marine waters and sediments. Consequently, its degradation is of significant environmental interest. Bacteria capable of growing on and potentially degrading plastic particles have been isolated from the Baltic Sea. Notable examples include *Rubripirellula* spp. (Kallscheuer et al., 2020), as well as various strains belonging predominantly to the phyla *Actinobacteria* and *Gammaproteobacteria* (Scales et al., 2021; Eronen-Rasimus et al., 2022). Conversely, the biotechnological production of biodegradable and environmentally friendly plastic represent a promising alternative (Aransiola et al., 2025; Multisanti et al., 2025). In this context, it is noteworthy that cold-adapted bacteria from the Baltic Sea, capable of producing polyhydroxyalkanoates, have been identified (Pärnänen et al., 2015). These biopolymers exhibit physicochemical properties similar to conventional plastics, and are therefore considered environmentally friendly materials. Although the specific bacterial producers were not identified, genetic analyses revealed the presence of *phaC* genes, which encode polyhydroxyalkanoate synthases (Pärnänen et al., 2015).

Lignin is a complex heteropolymer composed of alkyl and aromatic moieties. It is one of the most abundant biopolymers, occurring in the plant cell wall. *Pseudomonas* sp. strains capable of utilizing lignin-related compounds, including ferulate, *p*-coumarate, benzoate, and vanillin, have been isolated and characterised (Ravi et al., 2018). Depending on the growth medium, these bacteria excreted various usuful metabolites such as vanillyl alcohol, cis,cis-muconate, catechol, vanillate, and 4-hydroxybenzoate (Ravi et al., 2018).

The production of food using green technologies has emerged as a significant trend. Although plant cultivation remains an effective method of biomass production, it requires large areas of arable land and intensive irrigation, which presents increasing challenge due to limited water resources. Therefore, alternative biotechnological approaches are highly desirable. One promising strategy involves partial replacement of plant- and animal-derived proteins in the human diet with microbial proteins. Recently, a strain SoF1 of *Xanthobacter* sp. was isolated from the Baltic Sea (Klinzing et al., 2024). This autotrophic microorganism utilizes carbon dioxide as its sole carbon source and produces a protein-rich biomass that can be processed into a powder suitable for incorporation into various food products. Importantly, safety assessments have shown no evidence of genotoxicity or mutagenicity in the tested samples of this bacterial protein (Klinzing et al., 2024).

### 6.5 Assessing mutagenicity of marine water samples based on tests employing marine bacteria

Mutagenic pollution is another significant issue in the Baltic Sea and monitoring of this ecosystem for mutagenic pollutants is of critical importance. While numerous tests are available for the rapid assessment of mutagenicity in environmental samples, they rely on bacteria that do not thrive in marine waters, such as *Salmonella enterica* (Biran et al., 2010; Rueff et al., 2019; Dantas et al., 2020). Therefore, to enable effective and direct testing of the mutagenicity of marine water samples, an alternative assay was developed using the marine bacterium *Vibrio harveyi*, a species found in various marine habitats, including the Baltic Sea. Genetically modified *V. harveyi* strains were engineered to exhibit a high frequency of neomycin-resistant cells, which form colonies on nutrient agar containing antibiotics in the presence of low concentrations of mutagenic compounds (Czyz et al., 2000). Comparative studies demonstrated that this assay can detect lower concentrations of chemical mutagens than the traditional Ames test (Czyz et al., 2002). Furthermore, the *V. harveyi* assay was shown to be effective in detecting mutagenic pollution in Baltic Sea waters (Czyz et al., 2003). Subsequent studies led to the optimisation of assay conditions, further expanding its applicability. Another mutagenicity test employing the marine bacterium *V. harveyi* was also developed to use the bioluminescence phenomenon, characteristic of this species. Dark mutants of *V. harveyi,* bearing a point mutation in *luxE*, one of the genes responsible for light emission of bacterial cells, regained the ability to luminescence in the presence of mutagenic compounds due to the appearance of reverse mutations (Podgórska and Wegrzyn, 2006; Cheć et al., 2006).

Mutagenicity assays of biotechnological relevance, initially developed and optimized using marine bacteria, have since been applied in studies involving diverse marine samples, including water (Podgórska et al., 2007a), sediments (Podgórska et al., 2007b), and extracts from marine plants (Podgórska et al., 2008). A notable example includes the analysis of seawater from mussel farms, where the *Vibrio harveyi* bioluminescence assay was identified as “an optimal and rapid method for monitoring the water quality of mussel farms and as a tool for controlling the risks of pollution on mussel production and its safety for human consumption” (Ruiz et al., 2013). In another application, the assay was employed to assess the mutagenic, genotoxic, and cytotoxic activities of surface water samples from a wetland ecosystem in India (Kaur et al., 2014). Collectively, these studies underscore the broader applicability of biotechnological tools developed using marine bacteria, highlighting their potential for environmental monitoring and risk assessment across geographically and ecologically distinct regions.

In summary, Baltic Sea bacteria exhibit diverse biotechnological capacities that extend far beyond *Cyanobacteria*. Reported applications include the production of enzymes with unique properties, the degradation of complex organic pollutants, and the biosynthesis of bioactive compounds with pharmaceutical potential. Several taxa, particularly *Actinomycetes* and *Vibrio* species, stand out as prolific producers of bioactive molecules.

Other Baltic bacteria have been implicated in mineral cycling, plastic degradation, as well as bioplastic and microbial protein production. In addition, Baltic isolates have served as the basis for innovative environmental monitoring tools, such as mutagenicity assays employing genetically engineered and/or luminescent *Vibrio harveyi* strains, which offer higher sensitivity than conventional methods.

## 7 Biotechnological potential of Baltic fungi

**Figure 5:**
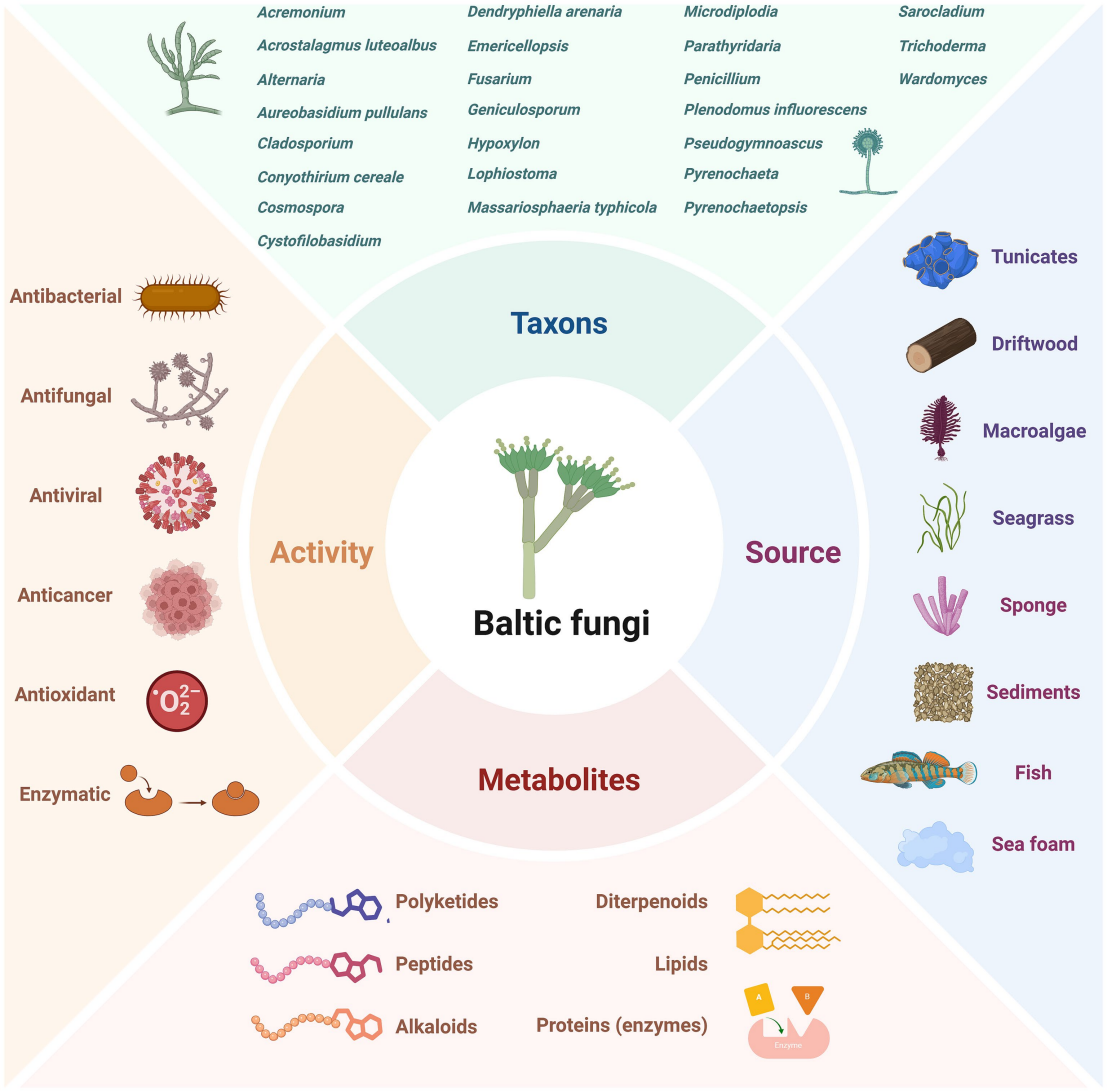
Diversity of investigated Baltic Sea fungi, their sources, produced metabolites and observed activities. The figure was created using BioRender.com.

The Baltic Sea harbours a variety of fungal species (Hassett et al., 2020; Tibell et al., 2020). However, compared to other microorganisms, knowledge of their true taxonomic diversity and environmental significance remains limited (Utermann et al., 2020a,b; Mazur-Marzec et al., 2024). These organisms have attracted the attention of researchers and biotechnologists due to the potential applications of their bioactive secondary products. In Baltic fungi, especially those living in symbiosis with aquatic animals and macrophytes or living in sediments, antimicrobial, anticancer, antiviral, and other activities have been observed (Figure 5).

**Figure 6:**
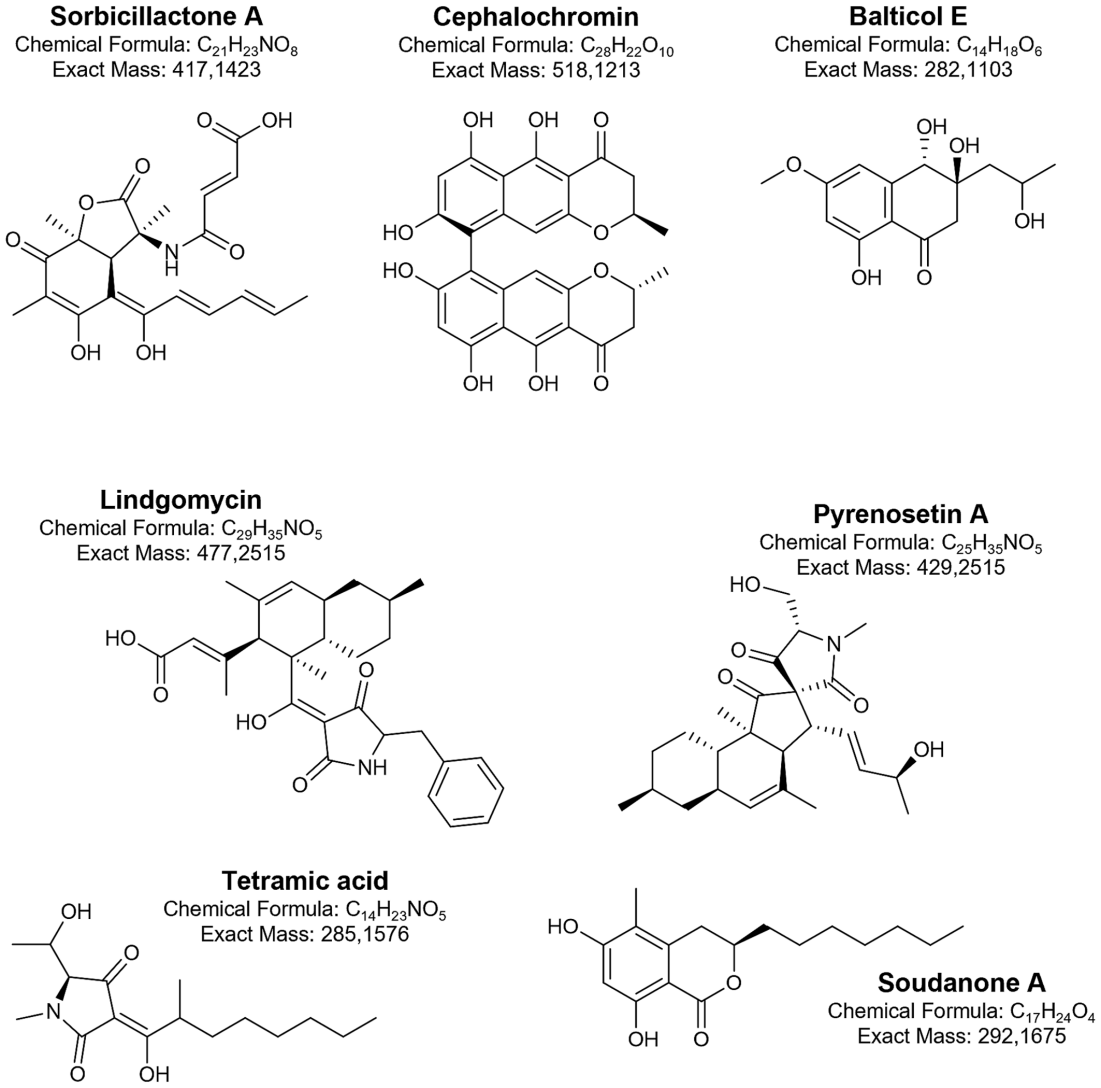
Biologically active metabolites produced by Baltic fungi.

In *Penicillium chrysogenum* strains from various sources, the presence of sorbicillin-derived alkaloids as well as sorbicillactones, was documented. This class of metabolites is also produced by the Baltic Sea fungi (Bringmann et al., 2007). The strain KIPB33 isolated from the Baltic sponge *Callopora aurita* showed a higher yield of sorbicillactone A (Figure 6) than other *P. chrysogenum* strains. Sorbicillactone A demonstrated highly selective activity against murine leukemic lymphoblast L5178y cells while exhibiting minimal cytotoxicity to healthy cells. To supply sufficient material for preclinical investigations, an efficient process for its large-scale biotechnological production was developed (Bringmann et al., 2007). Lindgomycin is another bioactive metabolite produced by sponge-associated Baltic fungi (Wu et al., 2015). Lindgomycin from *Massariosphaeria typhicola* displayed antimicrobial activity against different *Staphylococcus* strains.

Fungal isolates from the Baltic samples (water, sediment, seafoam, and plant debris) revealed the ability to interact with co-cultured phytopathogenic bacteria and fungi. In these cultures, interspecies communication was triggered, and an increase in chemical diversity was observed (Oppong-Danquah et al., 2018). The identified metabolites were classified as terpenes, alkaloids, peptides, and polyketides. In further studies, the large-scale co-cultivation of *Cosmospora* sp. with the phytopathogen *Magnaporthe oryzae supplied sufficient biomass for the* isolation and identification of several classes of metabolites, including pseudoanguillosporins A and B, naphtho-*γ*-pyrones, cephalochromin, and ustilaginoidin G, and ergosterol. Among other compounds, three known soudanones A, E, and D and two new soudanones variants, soudanones H and I, were detected (Figure 6) (Oppong-Danquah et al., 2022). Of these, soudanones E and D showed antimicrobial activity against *M. oryzae* and *Phytophthora infestans*, while pseudoanguillosporin A was active against *Pseudomonas syringae*, *Xanthomonas campestris*, *M. oryzae,* and *P. infestans*. In another co-culture experiment with sediment-derived fungi, *Plenodomus influorescens* and the phytopathogenic *Pyrenochaeta nobilis*, the anti-phytopathogenic activity of cephalochromin against *X. campestris* (IC₅₀ = 0.9 μg/mL) and *P. infestans* (IC₅₀ = 1.7 μg/mL) was revealed (Oppong-Danquah et al., 2020a). Moreover, fungi (*Penicillium* sp.) with activity against plant and human pathogens were also isolated from sea foam (Oppong-Danquah et al., 2020b). The most potent effects of extract from *Penicillium* strains were recorded in the case of *M. oryzae* and *Candida albicans* (IC₅₀ = 2.2 μg/mL and 6.3 μg/mL, respectively).

Another group of Baltic fungi, endophytes, primarily isolated from macroalgae, also gained attention for their promising bioactive properties. Tetramic acid sch210972 (Figure 6) produced by the endophytic ascomycete *Microdiplodia* sp. isolated from the green alga *Enteromorpha* sp. inhibits human leucocyte elastase (HLE) with an IC_50_ = 1.04 μg/mL, and shows moderate effects on the growth of *Bacillus megaterium* (Neumann et al., 2009). Generally, marine fungi, mainly from the genera *Penicillium*, *Cladosporium*, and *Aspergillus*, provide the majority of the known natural tetramic acid compounds (Jiang et al., 2020). The pharmacological potential of these compounds is related to their wide range of activities, including cytotoxic, antibacterial, and antifungal effects. From other endophytes, *Coniothyrium cereale* and *Wardomyces anomalus* OS4T3–2-1, cytotoxic phenalenones (Elsebai et al., 2011; Elsebai et al., 2012; Elsebai et al., 2016) and xanthone derivatives (Abdel-Lateff et al., 2003) were isolated.

From the brown algae *Fucus vesiculosus*, two endophytic fungal strains*, Pyrenochaetopsis* sp. FVE-001 and FVE-087, with anticancer activity, were isolated (Fan et al., 2020a,b). These strains were found to produce new decalinoylspirotetramic acid derivatives, pyrenosetins A-F (Figure 6), and the known decalin derivative—phomasetin. Pyrenosetins A and B demonstrated significant inhibitory activity against malignant melanoma A-375 cells, with IC₅₀ values of 2.8 and 6.3 μM, respectively (Fan et al., 2020a). Pyrenosetin D was toxic toward A-375 and HaCaT cells with IC_50_ values of 77.5 and 39.3 μM (Fan et al., 2020b). Pyrenosetin E inhibited the growth of A-375 with IC_50_ = 40.9 μM (Fan et al., 2022). *Geniculosporium* sp. 6,580, associated with the red algae *Polysiphonia* from the Baltic Sea, was cultured to yield 11 new botryane sesquiterpenoids (Krohn et al., 2005). Some of these compounds exhibited mild herbicidal, antifungal, and antibacterial activities. Their structures differed from known botryanes in substitution patterns and altered oxidation sites, alkylation, and unsaturation.

Extracts from fungal strains (mainly *Penicillium* species) isolated from the leaf and the root rhizosphere of Baltic eelgrass *Zostera marina*, showed strong anti-quorum sensing activity. They also displayed antimicrobial or anti-biofilm activity against Gram-negative environmental marine and human pathogens (Petersen et al., 2019). In later research (Tasdemir et al., 2024), the fungal (and bacterial) isolates from the microbiome of *Z. marina* were found to possess a wide spectrum of antipathogenic properties. The most active fungi, *Cladosporium halotolerans* and *Fusarium* sp., were linked to decaying *Z. marina* leaf surface. In the fungi, diketopiperazines, epipolythiodioxopiperazines, polyketides, diterpenoids, peptides, naphthoquinones, polyol lipids, and peptaibols were detected (Tasdemir et al., 2024). The analysis of three *Fucus* species with varying levels of fouling revealed that the least fouled *F. distichus* subsp. *evanescens* harbors abundant *Mucor* and *Alternaria*, known for producing antimicrobial and antifouling compounds, which may contribute to its fouling resistance (Oppong-Danquah et al., 2023).

In Baltic fungi collected from driftwood, six new naphthalenone derivatives (balticols A-F) (Figure 6), along with the known metabolite altechromone A, were isolated (Shushni et al., 2009; Kolařík et al., 2017). Among these, balticol E demonstrated the strongest antiviral activity against *Herpes simplex virus* type I (IC₅₀ 0.01 μg/mL). Balticolid, a novel class of 12-membered macrolide, active against antiHSV-1, was also identified (Shushni et al., 2011). Intrigued by its biological activity and interesting molecular architecture, an efficient approach to total balticolid synthesis from the known and cost-effective starting material was reported (Sudhakar et al., 2019). However, even if many *in vitro* tests with the application of these compounds were performed and showed promising results, limited data from *in vivo* experiments are available to date (Yi et al., 2020). Another fungus isolated from driftwood, *Lophiostoma*, produces oxasetin, an antibacterial polyketide that demonstrated *in vitro* activity against *Vibrio anguillarum*, *Flexibacter maritimus*, and *Pseudomonas anguilliseptica* (Shushni et al., 2013).

Some Baltic Sea fungi have been identified as degraders of shipwrecks (Björdal, 2012). Fungi involved in wood degradation produce cellulases, hemicellulases, and other enzymes capable of breaking down plant cell walls (Andlar et al., 2018). These enzymes are useful for producing biofuels, in the pulp and paper industry, and for converting agricultural waste into valuable products. Fucodainase was purified from the obligate marine fungus *Dendryphiella arenaria* TM94, isolated from Baltic Sea sand, and showed optimal activity at pH 6 and 50 °C (Wu et al., 2003). Fucoidanases can be involved in the production of sulfated fucooligosaccharides, compounds with a wide range of biological activities and pharmaceutical potential (Barzkar et al., 2023).

Fungi have been also studied for their ability to degrade and detoxify pollutants in marine environments. Research in the Baltic Sea suggests that the fungal communities within this ecosystem hold considerable potential for the bioremediation of petroleum hydrocarbons and other environmental pollutants (Al-Nasrawi, 2012; Miettinen et al., 2019). For example, diesel oil spills in the surface waters of the Baltic Sea increased the relative abundance of fungi from the phylum Ascomycota. The use of Baltic fungi as part of a hydrophobic sorbent for oil removal from water has also been tested (Paulauskiene et al., 2023).

Exposure experiments with polyethylene and polystyrene conducted in the Baltic Sea, with wood as a control surface, revealed that microplastic-associated fungal communities were distinct from those in the surrounding water and on wood (as a natural substrate) (Kettner et al., 2017). Understanding how fungal communities form and thrive on microplastics can provide valuable insights for developing methods for plastic waste degradation and advancing biofilm engineering.

Based on the studies discussed here, it can be concluded that fungi, as one of the least explored groups of microorganisms in the Baltic Sea, offer an opportunity to introduce innovative solutions that could both improve environmental conditions and contribute to better human health outcomes.

Baltic fungi represent an underexplored resource for biotechnology, offering diverse bioactive metabolites with pharmacological potential, industrially relevant enzymes, and promising applications in bioremediation and antifouling. While large-scale production, co-cultivation, and total synthesis demonstrate methodological feasibility, most findings remain limited to in vitro studies. Future research should prioritise in vivo validation, broader taxonomic and ecological exploration, and the systematic integration of biodiversity, functional traits, and metabolite profiling to unlock their full biotechnological potential.

## 8 Baltic viruses and their biotechnological potential

Reports on viruses from the Baltic Sea are relatively scarce. Among viruses infecting eukaryotic organisms, those specific to algae are most abundant, however, viruses pathogenic to insects, fish and seals have also been documented. The presence of viruses specific to pigs and humans strongly suggests the occurence of biological contaminants originating from urban or industrial environments. Nevertheless, to our knowledge, there are no reports on the biotechnological application of Baltic viruses specific to eukaryotes. Conversely, bacteriophages (or shortly – phages) are highly abundant viruses in the Baltic Sea (Mazur-Marzec et al., 2024). Their significant potential for biotechnological application is discussed below.

**Figure 7:**
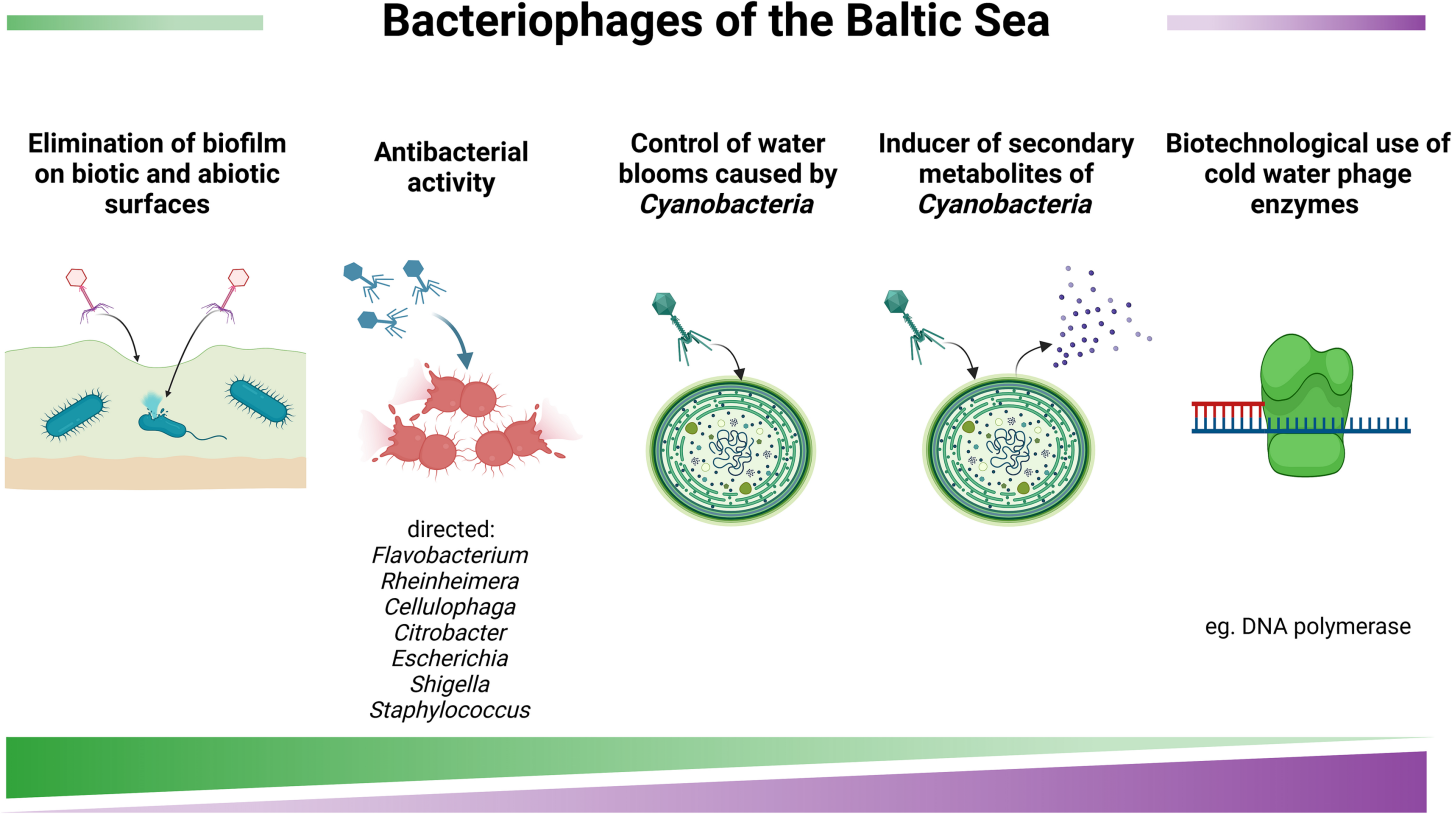
Baltic viruses and their biotechnological potential. The figure was created using BioRender.com.

One of the most prominent biotechnological application of phages is their use in combating pathogenic bacteria affecting various organisms (Figure 7). In the Baltic Sea, phages infecting members of the *Flavobacteriaceae* family are among the most abundant. Some of these bacteria are dangerous pathogens of marine animals, particularly fish (Nilsson et al., 2019). Therefore, bacteriophages could serve as effective agents for controlling such pathogens, especially in marine fish aquaculture systems (Daniel et al., 2021). Notably, phages specific to *Cellulophaga baltica* (Holmfeldt et al., 2007), *Flavobacterium* spp. (Nilsson et al., 2020; Daniel et al., 2021), and species from the genus *Rheinheimera* (Nilsson et al., 2019; Hoetzinger et al., 2021) have been identified in the Baltic Sea. In the addition to their potential use in aquaculture, bacteriophages may also help control bacterial infections in wild marine organisms. For example, the Baltic jellyfish *Aurelia aurita* is susceptible to infections caused by a variety of bacterial pathogens. Phages isolated from Baltic Sea water were shown to infect and efficiently lyse bacterial colonizers of jellyfish, including strains belonging to the genera *Citrobacter, Escherichia, Shigella* and *Staphylococcus* (Stante et al., 2024).

Although cyanobacteria are not direct pathogens to animals and humans, they can cause blooms and produce toxic compounds (Karlson et al., 2021). Therefore, it is potentially possible to use bacteriophages to control cyanobacterial blooms using specific cyanophages that would eliminate specific species of these microorganisms. Indeed, cyanophages were isolated from the Baltic Sea and characterised (Šulčius et al., 2015; Šulčius et al., 2019). As documented in previous section of this work, certain metabolites produced by cyanobacteria may have potential medical significance (Grabowski et al., 2024). However, large-scale production of such compounds is problematic, as the conditions that stimulate their synthesis are largely unknown. It was demonstrated that infecting cyanobacterial cells with cyanophages can enhance the production of various metabolites, including nodularin, anabaenopeptins, aeruginosins, and spumigins (Šulčius et al., 2018). Therefore, controlled cyanophage infection could be considered a potential method for stimulating the synthesis of such compounds.

Considering the antibacterial potential of bacteriophages, one of the interesting approaches may involve the use of phages with long tails in their virions. Such phages may prove particularly effective in combating biofilm-forming bacteria. Indeed, phages with these characteristics have been isolated from the Baltic Sea (Jakubowska-Deredas et al., 2012).

A fascinating group of viruses consists of phages isolated from the Baltic Sea ice. These phages infect psychrophilic hosts from the genera *Flavobacterium* and *Shewanella* and are capable of effectively replication within their host cells at temperatures as low as 3–4 °C (Luhtanen et al., 2014; Senčilo et al., 2015). Interestingly, the genomes of these psychrophilic bacteriophages encode various enzymes with potential biotechnological applications, including DNA polymerases, helicases, primases, DNA ligases, RNase H, and proteins involved in genetic recombination, such as homologues of NinB and NinG (Senčilo et al., 2015). Decreasing temperatures of various processes of *in vitro* DNA manipulation would be beneficial in genetic engineering. Thus, cloning corresponding genes from genomes of Baltic bacteriophages and overproduction and purification of their products might be of biotechnological significance.

Although research on viruses in the Baltic Sea is still less extensive than on other Baltic microorganisms, the available findings already highlight their considerable promise for biotechnology. Among them, bacteriophages are the most prominent group, with demonstrated potential to control bacterial pathogens relevant to aquaculture and marine ecosystems.

Cyanophages isolated from the Baltic Sea not only offer possibilities for mitigating harmful cyanobacterial blooms, but may also stimulate the production of valuable bioactive metabolites. Particularly intriguing are phages from Baltic Sea ice that thrive in low-temperature environments and encode enzymes, which could become powerful tools for genetic engineering and molecular biology under cold conditions.

## 9 Culture collections

Culture collections play a crucial role in preserving both living organisms and their genetic resources. They also provide organisms and their products for various areas of human activity, contributing to advancements in research, medicine and bioindustry (Garibay et al., 2024).

Effective management of these collections requires standardized protocols for cultivating organisms, detailed characterisation, and authentication of cultures, as well as the involvement of well-trained personnel. Furthermore, information about the source, taxonomic position, and ecological relationships of the preserved organisms should be accessible. In particular, if available, genomic data, are essential for identifying strains with biotechnological potential (Garibay et al., 2024).

In the Baltic countries, at least 79 culture collections of microorganisms have been identified, (Supplementary Table S3). In the living resources cathegory, microalgae (more than 100,000 isolates) and bacteria (more than 200,000 isolates) are the most abundant. However, it is challenging to estimate the contribution of Baltic Sea microorganisms in these collections, primarily due to insufficient characterisation and documentation of deposited strains. Another significant issue is the limited availability of online searchable catalogues, which hinders the broader utilization of the resources. Holdings of many research institutions remain uncatalogued, and thus, maintained microbial strains are used exclusively by local researchers. Improved documentation and cataloguing of these resources could significantly enhance their visibility, accessibility and value to the scientific community (Kurtzman and Labeda, 2009).

## 10 Genetic resources of Baltic microorganisms

There is a paradigm shift from traditional biology to bioinformatics that is revolutionizing exploitable biology driven by a rapid development of DNA sequencing and amplification technologies, annotation, proteome analysis, and phenotypic inventorying, resulting in the establishment of huge databases that can be mined in order to generate useful knowledge (Bull et al., 2000). Currently, sequencing technologies constitute a cost-effective and time-saving tool for sequencing microorganisms and samples that may harbor genes with potential biotechnological applications. Particularly, there is a remarkable increase in the number of microbial genomes retrieved from marine systems (e.g., Chen et al., 2024). There are numerous sequence databases, with the most well-known GenBank database,^1^ maintained by the National Center for Biotechnology Information (NCBI; a part of the National Institutes of Health in the United States) (Sayers et al., 2025), as part of the International Nucleotide Sequence Database Collaboration (INSDC). These developments are particularly relevant for microbial research, as microbial genomes and metagenomes are frequently used and redistributed via online databases, effectively decoupling physical access to genetic materials from downstream utilisation of sequence data. Consequently, the increasing availability of genomic data has raised important questions regarding access, benefit-sharing, and the governance of so-called digital sequence information (DSI). This issue has become a focal point in negotiations under the Convention on Biological Diversity (CBD) and its Nagoya Protocol, particularly during the adoption of the Kunming–Montreal Global Biodiversity Framework (GBF) in December 2022, which recognised the need for a multilateral mechanism to ensure the fair and equitable sharing of benefits arising from the use of DSI on genetic resources (CBD, 2022). The concrete design of this mechanism, including decisions on traceability, provenance metadata, and obligations for users and databases, remains under active development.

In the NCBI genome database, a total of 2,871,411 genomes are available, of which, 2,314,807 are annotated, and 39,268 are reference genomes.^2^ Due to a huge number of records, of which a large part lacked information on the isolation locations of the organism, we used the Bac*Dive* database to search for the genomes of Baltic Sea bacteria^3^ (Schober et al., 2025). The search with advanced options, using the geographical location ‘Baltic Sea’, yielded 70 results; however, genomic information was available only for 31 strains (Supplementary Table S4). Among 8,967 genomes assigned to the Cyanobacteriota/Melainabacteria group, there were 6231/2216/84/436 genomes assembled to levels of contig/scaffold/chromosome/complete genome. 8/1/14 genomes (scaffold/chromosome/complete genome level) belong to Baltic organisms.^4^ These genomes are not a comprehensive representation of the Cyanobacteria phylum: 14 represents the Nostocales order, 6 Synechococcales order (including one metagenome-assembled genome, MAG,) and one from the order Pleurocapsales. Also, two MAGs were obtained from unculturable cyanobacteria. The primary objectives of those genomic studies were (1) to identify gene clusters associated with bioactive metabolite production and, more broadly, to determine the biosynthetic potential of Baltic cyanobacteria, (2) to verify their taxonomy, (3) to determine adaptation to variable salinity conditions, (4) to identify host-microbiota interactions in holobiont (Voss et al., 2013; Teikari et al., 2018; Teikari et al., 2019; Österholm et al., 2020; Ahmed et al., 2021; Bonthond et al., 2021; Dreher et al., 2021; Heinilä et al., 2022). Baltic Cyanobacteria with available genomes are listed in Supplementary Table S5.

Among 29,056 genomes assigned to the Archaea kingdom there were 16,139/12058/98/761 genomes assembled to levels of contig/scaffold/chromosome/complete genome. To narrow down the search criteria, we took into account only representative genomes, with RefSeq category (738 genomes in total; with 252/118/126/360 genomes assembled to levels of contig/scaffold/chromosome/complete genome). Also, we checked among 2,604 MAGs, with 1477/450/24/653 assembled to levels of contig/scaffold/chromosome/complete genome. Unfortunately, none of them belong to Baltic organisms.^5^

Regarding the biodiversity of Baltic bacteriophages, the NCBI Database reports 199 bacteriophages isolated from various regions of the Baltic Sea with sequenced genomes.^6^ Among these, sixteen phages are classified as unassigned members of the *Caudoviricetes* class (Supplementary Table S6), while four phages are small, tailless members of the *Microviridae* family, characterised by ssDNA genomes of approximately 6 kb in size. The remaining 179 phages are classified into 20 genera within the *Caudoviricetes* class.

As reported by Šulčius and Holmfeldt (2016), phages that infect *Cellulophaga baltica* (formerly known as *Flavobacterium aceae*) belong to the following genera: *Akihdevirus, Bacelvirus, Baltivirus, Callevirus, Cbastvirus, Cebadecemvirus, Cellubavirus, Helsingorvirus*, and *Lightbulbvirus*. These phages possess dsDNA genomes ranging from 38.5 kb to 145.9 kb (Holmfeldt et al., 2007; Šulčius and Holmfeldt, 2016). The largest collection of phages infecting *Rheinheimera* sp. was created by Nilsson et al. (2019), comprising 54 phages from the *Barbavirus* genus, with genomes ranging from 80.2 kb to 84.6 kb.

The majority of Baltic phages with sequenced genomes (over 100) infect *Flavobacterium* spp., and more than half of these belong to the *Elemovirus* genus, with genomes around 60 kb. Other abundant genera include *Muminvirus* (genomes of 37.5 kb to 38.5 kb) and *Lillamyvirus* (genomes of 37.5 kb to 39 kb) (Nilsson et al., 2020; Hoetzinger et al., 2021). The largest genomes among Baltic Flavobacterium phages (~166 kb) belong to the *Immutovirus* genus (Hoetzinger et al., 2021).

The only cyanophage from the Baltic Sea with a sequenced genome in the NCBI database is the vB_AphaS-CL131 virus, which infects *Aphanizomenon flos-aquae* (Šulčius et al., 2015; Šulčius et al., 2019). Recently, one of phages isolated from Kiejl Fjord infects *Staphylococcus* sp. and is currently the only example of a Baltic virus specific to *Bacillota* (Stante et al., 2024). Among the unassigned members of the *Caudoviricetes* class, *Shewanella* viruses were also identified. Interestingly, the isolation and cultivation of ice bacteria and cold-active phages from the Baltic Sea ice were reported (Luhtanen et al., 2014; Senčilo et al., 2015).

## 11 Rules of access to microbial resources of the Baltic Sea in the context of the Nagoya protocol

### 11.1 International law

Researchers and organizations (institutions, companies, etc.) conducting research and accessing microbial resources must adhere to international and national regulations. Many countries require compliance with these regulations to achieve intellectual property protection. A lack of awareness or non-compliance may jeopardize their research and innovation outcomes (Schneider et al., 2022). Moreover, there has been some debate about whether publishers should mandate documentation of compliance with the Nagoya Protocol for manuscript submissions dealing with genetic resources (Kumar, 2018).

**Figure 8:**
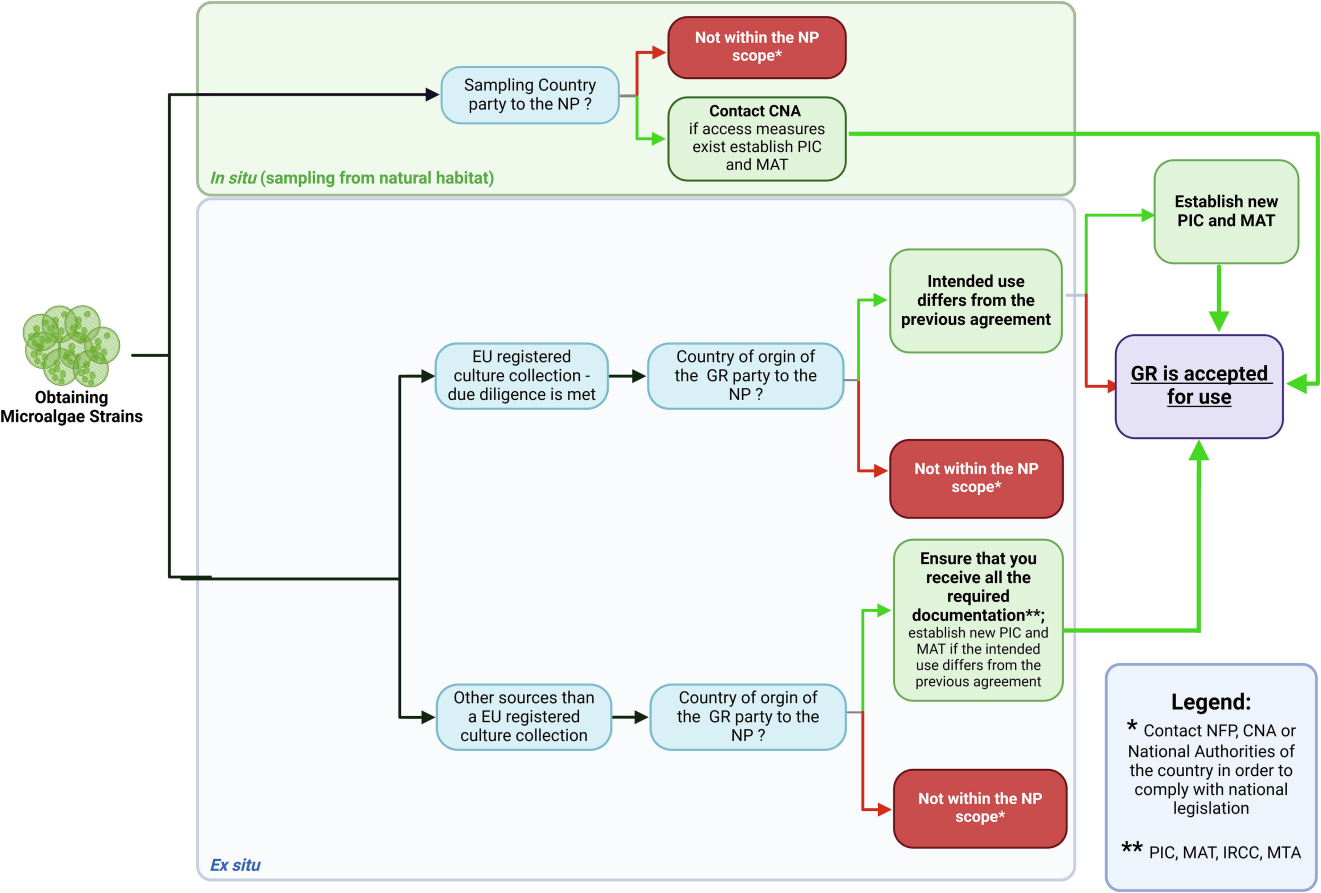
Decision framework proposed by Martins et al. (2020) presenting the steps necessary to obtain access to genetic resources (GR) according to Nagoya Protocol (NP). The figure was created using BioRender.com.

Researchers need to be aware that, as reaffirmed by the Convention on Biological Diversity (CBD),^7^ adopted in 1992 at the Rio Earth Summit, States have sovereign rights over their biological resources within their jurisdiction. In the case of coastal countries, the right to explore, exploit, conserve and manage natural resources also includes marine waters, the seabed and its underground of the country’s territorial waters, and extends to the exclusive economic zone, up to 200 nautical miles from the baseline as stated by the 1982 United Nations Convention on the Law of the Sea.^8^ This means that each state has the authority to regulate and manage its biological resources in accordance with its laws and policies. This includes controlling access to these resources, setting conditions for their use, and establishing mechanisms for sharing the benefits derived from their utilization. Anyone seeking to access genetic resources from a country of origin must obtain prior informed consent (PIC) from the relevant authorities (unless the country has specified otherwise) and establish mutually agreed terms (MAT) for their use (Figure 8).

Based on these principles, another international agreement, the Nagoya Protocol on Access to Genetic Resources and the Fair and Equitable Sharing of Benefits Arising from their Utilization,^9^ was adopted in 2010.

Under this regulation, commonly referred to as ABS regulation (Access and Benefit Sharing), all countries party to the NP are required to designate (i) an ABS National Focal Point (NFP) responsible for providing information on how to obtain the PIC and establish MAT, and (ii) Competent National Authorities (CNA), which grant access to genetic resources (Figure 8). Additionally, countries must make their legislative, administrative, and policy measures related to ABS available through a searchable database called the ‘ABS Clearing House.’^10^ This website should be a starting point for anyone seeking access to bioresources. However, it is important to note that even countries that have not signed or implemented the NP may have national legislation governing access to genetic resources and ABS.

While a detailed overview of the NP is beyond the scope of this paper, it is worth mentioning that the NP broadly defines genetic resources (GR). It includes (i) any material of plant, animal, microbial or other (non-human) origin containing functional units of heredity that can be explored through research and utilized in development, thus having actual or potential value, as well as (ii) their derivatives—naturally occurring biochemical compounds resulting from genetic expression or metabolism, such as proteins (e.g., enzymes), lipids, RNA, or secondary metabolites.

Utilization refers to the use, exploration, research and/or development of GR. It is important to note that this term encompasses basic research, and many research activities—regardless of whether they have a commercial component—fall within the scope of ABS regulation, as they generate new knowledge and may, in the future, lead to the development of new commercial products. While some national ABS laws differentiate between non-commercial and commercial use (e.g., with varying benefit-sharing requirements), it is a common misconception that NP applies solely to commercial research. Another misunderstanding is that benefit-sharing is limited to monetary benefits. In fact, the NP also foresees non-monetary benefits, such as data sharing, joint publications, project partnerships, education, training, and shared reference collections-practices with a long-standing tradition in academia. In addition to the CBD and the Nagoya Protocol, it is also worth mentioning recent legal developments concerning marine biological diversity in areas beyond national jurisdiction (BBNJ). These regions, often referred to as the “high seas,” lie outside the sovereignty of individual States and until recently lacked a comprehensive legal framework for access and benefit-sharing of marine genetic resources. In June 2023, the United Nations adopted the *Agreement under the United Nations Convention on the Law of the Sea on the conservation and sustainable use of marine biological diversity of areas beyond national jurisdiction* (the “BBNJ Treaty”), introducing rules for access, use, and fair sharing of benefits arising from such resources.^11^ The BBNJ Treaty will enter into force on 17 January 2026, 120 days after the 60th instrument of ratification is deposited. To ensure coherent implementation in Europe, the European Commission has proposed a Directive of the European Parliament and of the Council on the conservation and sustainable use of marine biological diversity of areas beyond national jurisdiction, designed to transpose the BBNJ Treaty into EU law and extend ABS principles into the high seas governance regime.^12^

### 11.2 The Baltic Sea case

The Baltic Sea, surrounded by nine countries: Denmark, Germany, Poland, Lithuania, Latvia, Estonia, Finland, Sweden and Russia, is a confined sea where opposite coasts are never more than 400 nautical miles apart, and most of the maritime boundaries have been settled (Franckx, 2021). Depending on their geographic origin, Baltic microbial resources fall under the jurisdiction of either of the bordering countries. While all Baltic-bordering countries are Parties to the CBD (at the time of writing), only five—Denmark, Estonia, Finland, Germany and Sweden are Parties to the NP (Supplemntary Table S7).

However, since the European Union (EU) ratified the protocol on May 16th 2014 (EU, 2014), it became a Party to the NP, meaning that all EU member states, as well as users of GR and associated traditional knowledge (aTK) within the EU, must comply with EU regulations on ABS (EU ABS, 2014). The main objectives of these two adopted documents, the ABS Regulation (EU No 511/2014) and the Implementing Regulation (EU, 2015), are to prevent the utilization of the GR or aTK in the Union that were not accessed in accordance with the requirements of NP, to promote fair and equitable benefit-sharing, and to ensure legal certainty regarding the use of these resources. Researchers within the EU who use genetic resources are now legally obliged to follow specific procedures, including obtaining permits, maintaining records, and submitting due diligence declarations. An exemplary decision framework proposed by Martins et al. (2020) presents some initial steps necessary to obtain microalgae GR.

To assist users in navigating the complexities of ABS law, the European Commission published a guidance document (Guidance Document, 2021). While not legally binding, it serves as a useful reference for clarifying many of the intricate issues arising from ABS regulations.

However, the decision to regulate access to their GR under the NP remains within the competence of individual Member States. Fortunately, for those wishing to utilize Baltic genetic resources, some countries bordering the Baltic Sea have chosen not to assert sovereign rights over their genetic resources under the NP, thus reducing administrative burdens. This means that, in practice, these resources are freely accessible, and there is no requirement to establish Prior Informed Consent or negotiate Mutually Agreed Terms for their utilization.

Nevertheless, users who obtain microbial resources *in situ* may need to comply with other national legislation, such as those related to protected areas, which should be considered before sampling the country’s sovereign genetic resources. An overview of access and benefit-sharing policies in nine Baltic coastal countries, along with their NP status, is summarised in Supplementary Table S7. The relevant information for each country can be found on the ‘ABS Clearing House’ website,^13^ while details on access rules are available in the countries’ Interim National Reports on the Implementation of the Nagoya Protocol.

Russia, the only non-EU country in the Baltic Sea, is not a Party to NP; thus, the utilisation of GR originating in Russia is outside the scope of the EU ABS Regulation. However, the EU recommends that users of such resources should comply with the country’s national legislation or regulatory requirements and respect any mutually agreed terms entered into.

### 11.3 Obtaining the Baltic genetic resources ex-situ

The NP and EU ABS regulations also apply to genetic resources (e.g., strains, specimens) from culture collections held in scientific institutions. Within the EU, users accessing material from these collections (access of GR) are required to seek, obtain, and maintain the relevant information (such as PIC, MAT), transfer it to the subsequent users (exercising due diligence), and submit a due diligence declaration at a designated checkpoint (either at the research funding stage or upon the final development of a product), as specified in the Implementing Regulation (EU) 2015/1866 (Article 7). To facilitate compliance and reduce administrative requirements, the European Commission (EC) has established a voluntary Register of Collections within the Union. Collections are granted registered status upon verification by the relevant national competent authority that they meet the criteria specified in Regulation (EU) No 2015/1866. Users accessing genetic resources from the registered collection are deemed to have exercised due diligence in obtaining all necessary information. At the time of writing, three collections have been recognised, as recognized through a Commission Decision.^14^

Accessing genetic resources from collections originating in countries that have chosen not to impose access regulations does not create ABS obligations on the user. Since most Baltic-bordering countries allow grants of free access to their GR, culture collections in the region can provide these resources without the required PIC and MAT.

## 12 Future perspectives

Despite the discovery of many promising avenues for the biotechnological application of Baltic Sea microorganisms, the successful commercialisation of their potential depends on addressing several key challenges. These include the limited amount of biomass obtained from organisms collected from the environment or grown in laboratory cultures, insufficient quantities of isolated compounds for extensive studies, and suboptimal physicochemical or biological properties of bioactive compounds. Additional obstacles include difficulties in securing long-term funding for basic research and limited interest from the industrial sector in developing novel bioproducts.

Today, modern scientific tools offer powerful solutions to these early-stage challenges. Artificial intelligence (AI)-based technologies and comprehensive natural product databases are playing an increasingly important role in accelerating the discovery pipeline. However, to date, these tools have been used to a limited extent in studies of biotechnological potential of Baltic microorganisms. Meanwhile, by applying suitable AI algorithms, it is now possible to conduct high-throughput screening of large datasets, enabling rapid identification of compounds with properties optimised for specific applications. These tools also allow *in silico* prediction of biological activity and pharmacokinetic properties, significantly reducing the time and resources needed in the discovery phase. Furthermore, systems biology approaches enhance our understanding of microbial metabolism, physiology, and the genetic basis of their biosynthetic capabilities. In parallel, advances in synthetic biology and chemical synthesis offer the means to produce natural products with improved characteristics, in quantities sufficient to support both in-depth research and commercialisation. The integration of these modern tools not only streamlines the development of new natural products, but also increases the likelihood of selecting compounds with high application potential. Nevertheless, the ultimate success of such endeavours depends on close collaboration between the scientific community and industry stakeholders. Strengthening these partnerships will be essential to unlocking the full biotechnological value of microorganisms from the Baltic Sea and other ecosystems.

## Footnotes

^1^National Center for Biotechnology Information. https://ncbi.nlm.nih.gov/ (accessed 28 February 2025).

^2^National Center for Biotechnology Information – Genome Datasets. https://ncbi.nlm.nih.gov/datasets/genome/ (accessed 28 February 2025).

^3^The Bacterial Diversity Database BacDive. https://bacdive.dsmz.de/ (accessed 28 February 2025).

^4^National Center for Biotechnology Information – Cyanobacteriota/Melainabacteria Genomes. https://ncbi.nlm.nih.gov/datasets/genome/#!/prokaryotes/ (accessed 28 February 2025).

^5^National Center for Biotechnology Information – Cyanobacteriota/Melainabacteria Genomes. https://ncbi.nlm.nih.gov/datasets/genome/#!/prokaryotes/ (accessed 28 February 2025).

^6^National Center for Biotechnology Information – Genome list. https://ncbi.nlm.nih.gov/ (accessed 28 February 2025).

^7^The convention on biological diversity. https://cbd.int/doc/legal/cbd-en.pdf (accessed 6 June 2023).

^8^United Nations convention on the law of the sea. https://un.org/depts/los/convention_agreements/texts/unclos/unclos_e.pdf (accessed 6 June 2023).

^9^The Nagoya Protocol on access to genetic resources and fair and equitable sharing of benefits arising from their utilization. https://www.cbd.int/abs/doc/protocol/nagoya-protocol-en.pdf (accessed 6 June 2023).

^10^Access and Benefit Sharing Clearing House. https://absch.cbd.int/en/ (accessed 6 June 2023).

^11^United Nations rules for access, use, and fair sharing of benefits from marine biological diversity of areas beyond national jurisdiction. https://www.un.org/bbnj (accessed 1 October 2025).

^12^Proposal for a Directive of the European Parliament and of the Council on the conservation and sustainable use of marine biological diversity of areas beyond national jurisdiction (Document 52025PC0173). https://eur-lex.europa.eu/legal-content/EN/TXT/?uri=celex:52025PC0173 (accessed 1 October 2025).

^13^Access and Benefit Sharing Clearing House. https://absch.cbd.int/en/ (accessed 6 June 2023).

^14^European Commission Decision on the collections recognition. https://environment.ec.europa.eu/topics/nature-and-biodiversity/sharing-natures-genetic-resources_en (accessed 6 June 2023).
