# Supplementary material for: Unlocking the biotechnological potential of Baltic microorganisms
Source: Front Microbiol. 2025 Oct 28;16:1682611. doi: 10.3389/fmicb.2025.1682611 (PMC12602537; doi:10.3389/fmicb.2025.1682611)
Supplement: Supplementary file 1 [file Data_Sheet_1.docx]

Unlocking the Biotechnological Potential of Baltic Microorganisms

Hanna Mazur-Marzec ^1,*^, Łukasz Grabowski ^2^, Alicja Węgrzyn ^3^, Agata Błaszczyk ^1^, Marta Cegłowska ^4,*^, Przemysław Dąbek ^5^, Momina Farooq ^1^, Ewa Górecka ^5^, Agata Jurczak-Kurek ^6^, Anna-Karina Kaczorowska ^7^, Tadeusz Kaczorowski ^8^, Marija Kataržytė ^9^, Robert Konkel ^1^, Ewa Kotlarska ^10^, Donata Overlingė ^9^, Waldemar Surosz ^1^, Anna Toruńska-Sitarz ^1^, Semko Walat ^1^, Monika Zielenkiewicz ^2^, Grzegorz Węgrzyn ^2^

^1^Department of Marine Biology and Biotechnology, University of Gdańsk, Gdynia, Poland

^2^Department of Molecular Biology, University of Gdańsk, Gdańsk, Poland

^3^University Center for Applied and Interdisciplinary Research, University of Gdańsk, Gdansk, Poland

^4^Department of Marine Chemistry and Biochemistry, Institute of Oceanology, Polish Academy of Sciences, Sopot, Poland

^5^Institute of Marine and Environmental Sciences, University of Szczecin, Szczecin, Poland

^6^Department of Evolutionary Genetics and Biosystematics, University of Gdańsk, Gdansk, Poland

^7^Collection of Plasmids and Microorganisms, University of Gdańsk, Gdansk, Poland

^8^Laboratory of Extremophiles Biology, Department of Microbiology, University of Gdańsk, Gdańsk, Poland

^9^Marine Research Institute, Klaipeda University, Klaipeda, Lithuania

^10^Department of Genetics and Marine Biotechnology, Institute of Oceanology, Polish Academy of Sciences, Sopot, Poland

^11^Department of Phycology, University of Gdańsk, Gdynia, Poland

*** Correspondence: * Correspondence:**Hanna Mazur-Marzec
hanna.mazur-marzec@ug.edu.pl
Marta Cegłowska
mceglowska@iopan.pl

**Table S1.** Biotechnology companies operating in the Baltic Sea region and utilizing microalgae.

| **Company (Country)** | **Microorganism used** | **Products/Application** | **Website** |
| --- | --- | --- | --- |
| Biotrino APS (Denmark) | *Chlorella vulgaris* | Food supplements | https://biotrino.com |
| Biovento (Poland) | 12 strains of benthic and aggregating Baltic Sea microalgae | Maritime industry – antifouling paint | https://biovento.wixsite.com/biovento |
| Ekogina (Lithuania) | *Arthtrospira platensis* and *Chlorella vulgaris* | Food supplements | https://powdi.lt |
| Furcella OÜ (Estonia) | *Haematococcus pluvialis* | Cosmetics with Astaxanthin – component of skin care products | https://berrichi.eu |
| Simris Biologics (Sweden) | Library of over 1200 strains of cyanobacteria producing over 5000 novel compounds | Cyanotoxin standards and reagents  Antibody Drug Conjugates (ADC) – cyanobacterial non-ribosomal peptides (not available on the market) | https://simrisbiologics.com |
| Spila (Lithuania) | *Arthrospira platensis* and *Chlorella vulgaris* | Food supplements,  Food additives | https://spila.lt |
| Spirulina Nord (Latvia) | *Arthrospira platensis* | Food supplements | https://spirulinanord.eu |

**Table S2.** Selected biotechnological projects implemented with the participation of the Baltic Sea region countries, using marine organisms. The projects were funded by the EU framework programs: FP7 (2007-2013), FP8/Horizon 2020 (2014-2020), and FP9/Horizon Europe (2021-2027).

| Country | Project Tittle | Acronym | Overall budget (€) | Years of implementation | Website |
| --- | --- | --- | --- | --- | --- |
| FP7 | | | | | |
| Finland, Poland, Sweden | Exploring Marine Resources for Bioactive Compounds: From Discovery to Sustainable Production and Industrial Applications | MAREX | 7 895 271,64 | 2010-2014 | https://cordis.europa.eu/project/id/245137 |
| Germany, Finland | Natural Products from Marine Fungi for the Treatment of Cancer | MARINE FUNGI | 3 850 906,59 | 2011-2014 | https://cordis.europa.eu/project/id/265926 |
| Germany | FUME (Functional Metagenomics – Harnessing the Biotechnological Potential of Completely Novel Protein Families) | FUME | 1 499 441,60 | 2011-2016 | https://cordis.europa.eu/project/id/281384 |
| Germany, Poland | New Biotechnological Approaches for Biodegrading and Promoting the Environmental Biotransformation of Synthetic Polymeric Materials | BIOCLEAN | 3 925 096,90 | 2012-2015 | https://cordis.europa.eu/project/id/312100 |
| Germany | Marine Microbial Biodiversity, Bioinformatics, Biotechnology | MicroB3 | 11 496 409,35 | 2012-2015 | https://cordis.europa.eu/project/id/287589 |
| Danmark, Germany | Marine Microorganisms: Cultivation Methods for Improving their Biotechnological Applications | MACUMBA | 11 970 227,60 | 2012-2016 | https://cordis.europa.eu/project/id/311975 |
| HORIZON2020 | | | | | |
| Germany, Sweden | Sustainable Exploitation of Bio-based Compounds Revealed and Engineered from Natural Sources. | SECRETed | 7 787 818,75 | 2021-2025 | https://cordis.europa.eu/project/id/101000794 |
| Denmark, Germany | Marine Biodiversity as Sustainable Resource of Disease-Suppressive Microbes and Bioprotectants for Aquaculture and Crop Diseases | MARBLES | 7 496 896,25 | 2021-2026 | https://cordis.europa.eu/project/id/101000392 |
| HORIZON EUROPE | | | | | |
| Estonia, Finland, Denmark, Germany, Lithuania Poland, Sweden | LOCALITY - Nature-Positive Algae-Based Food, Agriculture, Aquaculture and Textile Products Made in North and Baltic Sea Ecosystems | LOCALITY | 9 819 477,50 | 2023-2027 | https://cordis.europa.eu/project/id/101112884 |
| Estonia, Denmark, Finland, Germany, Latvia, Sweden | Accelerating Algae Product Developments in Baltic and North Sea | AlgaePro BANOS | 12 027 291,25 | 2023-2027 | https://cordis.europa.eu/project/id/101112943 |
| Lithuania | InnoAerogel: Sustainable Sorbent for Aquatic Oil Spills Clean-Up | INNOAEROGEL | 2 294 720,00 | 2024-2026 | https://cordis.europa.eu/project/id/101188620 |

**Table S3.** Culture Collections based in the Baltic Sea Region.

| **Country** | **City** | **Full Name** | **Acronym** | **Total number of strains** | **Organizational member** | **website** |
| --- | --- | --- | --- | --- | --- | --- |
| Denmark | Copenhagen | Natural History of Denmark Collections |  | 400000 |  | https://samlinger.snm.ku.dk/en/dry-and-wet-collections/botany/algal-herbarium/ |
|  |  | The International Escherichia and Klebsiella Centre (WHO) | IEKC | 63500 | WDCM | https://en.ssi.dk/surveillance-and-preparedness/national-reference-laboratories/escherichia-shigella-og-klebsiella |
|  | Kongens Lyngby | IBT Fungal Culture Collection | IBT | 46500 | WDCM, ECCO | https://bioengineering.dtu.dk/research/strain-collections/ibt-culture-collection |
| Estonia | Tartu | Estonian Yeast Stock Collection | 101TYVE | 2900 | WDCM |  |
|  |  | BioCC Microbial Collection | BioCC-MC | 1315 | WDCM, WFCC, ECCO | https://eccosite.org/portfolio-item/biocc-mc/ |
|  |  | Collection of Environmental and Laboratory Microbial Strains | CELMS | 2000 | WDCM, ECCO | https://eemb.ut.ee/eng/celms_english_introduction_list.php |
|  |  | Collection of Reproductive Tract Microorganisms | CREP | 2050 | WDCM | https://eemb.ut.ee/humb_eesti_Contacts_list.php |
|  |  | WFCC Estonian Human Microbiota Biobank | HUMB | 22047 | WDCM, ECCO | https://eemb.ut.ee/humb_eesti_Contacts_list.php |
|  |  | Tartu Fungal Culture Collection | TFC | 3000 | WDCM | https://kogud.emu.ee |
| Finland | Espoo | VTT Technical Research Centre of Finland Ltd. | VTT*** | 6579 | WDCM, WFCC, ECCO | https://culturecollection.vtt.fi/ |
|  | Helsinki | Hambi Culture Collection | HAMBI | 4278 | WDCM, WFCC, ECCO | https://helsinki.fi/en/infrastructures/biodiversity-collections/infrastructures/microbial-domain-biological-resource-centre-hambi |
|  |  | Marine Research Laboratory at Syke Culture Collection |  | 100 |  | https://finmari-infrastructure.fi/facilities/laboratories/syke-mrc-marine-ecology-laborato/ |
| Germany | Braunschweig | Institut für Pflanzenschutz im Forst | BBLF | 500 | WDCM |  |
|  |  | Leibniz-Institut DSMZ-Deutsche Sammlung von Mikroorganismen und Zellkulturen Gmbh | DSMZ*** | 33784 | GCM, WDCM, WFCC, ECCO | https://dsmz.de |
|  | Essen | Central Collection of Algal Cultures | CCAC | 2060 | WDCM, WFCC, ECCO | https://uni-due.de/biology/ccac/ |
|  | Freising | Weihenstephan Microbial Strain Collection | WS, WSBC, WSLC, WSYC, WSMC | 10600 | WDCM | https://iel.tum.de/ziel/ueber-uns/alumni/ziel-abteilung-mikrobiologie-lehrstuhl-fuer-mikrobielle-oekologie/microbial-strain-collection/ |
|  | Göttingen | The Sammlung von Algenkulturen der Universität Göttingen | SAG | 2269 | WDCM, WFCC, ECCO | https://epsag.uni-goettingen.de/ |
|  | Hamburg | Microalgae and Zygnematophyceae Collection Hamburg | MZCH-SVCK | 714 | WDCM, WFCC | https://mzch-svck.uni-hamburg.de/ |
|  | Hannover | Institute of Virology | MHH | 120 | WDCM |  |
|  |  | Nationales Konsiliarlabor für Adenoviren | NKA | 2 | WDCM |  |
|  | Hustedt | Hustedt Diatom Collection |  |  |  | https://awi.de/en/science/biosciences/polar-biological-oceanography/main-research-focus/hustedt-diatom-study-centre/diatom-collection.html |
|  | Jena | Jena Microbial Resource Collection | JMRC | 50011 | GCM, WDCM | https://leibniz-hki.de/de/jena-microbial-resource-collection.html |
|  | Kiel | Institut für Allgemeine Mikrobiologie | IFAM | 1536 | WDCM |  |
|  |  | Streptokokken Sammlung | IFBM | 500 | WDCM |  |
|  | Marburg | Medical Culture Collection Marburg | MCCM | 3000 | WDCM |  |
|  | Potsdam | CCCryo - Culture Collection of Cryophilic Algae | CCCryo | 987 | GCM, WDCM, ECCO | https://cccryo.fraunhofer.de/ |
|  | Valley | Collection of Building Relevant (Micro)Organisms | BRMO | 460 | WDCM |  |
|  | Würzburg | Bayerische Landesanstalt für Weinbau und Gartenbau | BLWG | 900 | WDCM |  |
| Latvia | Riga | Microbial Strain Collection of Latvia | MSCL*** | 1452 | WDCM, ECCO | https://mikro.daba.lv |
| Lithuania | Vilnius | Collection of Microorganisms Nature Research Centre |  | 233 |  |  |
| Norway* | Oslo | Medical Microbiological Laboratory | MML | 1910 | WDCM |  |
|  |  | The Norwegian Culture Collection of Algae, NORCCA | NORCCA | 1118 | WDCM, ECCO | https://norcca.scrol.net |
| Poland | Gdańsk | Collection of Plasmids and Microorganisms | KPD*** | 661 | GCM, WDCM, ECCO | https://kpd.ug.edu.pl |
|  |  | Collection of Salmonella Microorganisms | KOS | 1974 | WDCM, ECCO |  |
|  | Szczecin | Szczecin Collection of Diatoms | SZCZ | 2700 |  | https://www.facebook.com/marinebenthicdiatomproject/ |
|  |  | Szczecin Diatomological Collection | SZCZ | 30000 | GBIF Registry | https://herbariumpomeranicum.pl |
|  | Gdynia | Culture Collection of Baltic Algae | CCBA | 230 | WDCM | https://ccba.ug.edu.pl |
|  |  | Culture Collection of Northern Poland | CCNP |  |  |  |
|  | Lódź | Centre of Industrial Microorganisms Collection | LOCK | 632 | WDCM, WFCC |  |
|  | Olsztyn | Culture Collection at University of Warmia and Mazury in Olsztyn | LCC | 297 | WDCM |  |
|  | Poznań | Collection of Plant Pathogens | CPPIPP | 889 | WDCM |  |
|  | Puławy | Department of Microbiology, Veterinary Branch of National Strain Collection | DMVB | 450 | WDCM |  |
|  | Słupsk | Herbarium Pomeranicum |  | 470225 |  | https://herbariumpomeranicum.pl |
|  | Sopot | Culture Collection of Microorganism IO PAN | CCM | 30 |  | https://old.iopan.pl/BioChem/collections.html |
|  |  | IOMB Strain Collection IO PAN | IOMB |  |  | https://old.iopan.pl/MolBio/IOMB.html |
|  | Warszawa | Collection of Industrial Microorganisms | IAFB*** | 1165 | WDCM, ECCO | https://cim.ibprs.pl/ |
|  |  | Collection of Microorganisms Producing Antibiotics | IBA | 708 | WDCM, ECCO |  |
|  | Wrocław | Polish Collection of Microorganisms | PCM*** | 3250 | WDCM, WFCC, ECCO | https://pcm.org.pl/home |
| Russia** | Krasnodar | Collection of Eubiotic and Epiphytic Microorganisms | CEEM*** | 160 | WDCM | https://mb.kubsau.ru/ |
|  | Moscow | All-Russian Collection of Microorganisms | VKM*** | 18765 | GCM, WDCM, WFCC, ECCO | https://vkm.ru/index.htm |
|  |  | Collection of Bacteriophages and Microorganisms Micromir | CBMW | 3180 | WDCM |  |
|  |  | Collection of Microalgae, Department of Mycology and Algology, Faculty of Biology, MSU | MSUALGDMA | 67 | WDCM |  |
|  |  | Culture Collection of Microalgae and Cyanobacteria IPPAS | IPPAS | 421 | WDCM, ECCO | https://cellreg.org/Catalog/ |
|  |  | Culture Collection of Wild and Cultivated Macromycetes | MSU_FM | 44 | GCM, WDCM | https://mycol-algol.ru/collection_2.html |
|  |  | Russian National Collection of Industrial Microorganisms | VKPM | 15250 | WDCM, WFCC, ECCO | https://vkm.ru/collecti.htm |
|  |  | The Russia Research Institute for Antibiotics Culture Collection | RIA | 2069 | WDCM |  |
|  |  | Yeast Collection of the Soil Biology Department in Lomonosov Moscow State University (KBP MSU or KBP) | KBP MSU | 1800 | WDCM | https://depo.msu.ru |
|  | Murmansk | The Collection of Cyanoprokaryotes of The Polar-Alpine Botanic Garden-Institute | KPABG | 215 | WDCM | https://isling.org/cyano |
|  | Perm | Regional Specialized Collection of Alkanotrophic Microorganisms | IEGM | 2014 | GCM, WDCM, WFCC, ECCO | https://iegm.ru/iegmcol/index.html |
|  | Pushchino | Algal Collection of Soil Science Institute | ACSSI | 435 | WDCM |  |
|  | Saratov | Collection of Rhizosphere Microorganisms | IBPPM | 235 | WDCM, WFCC | https://collection.ibppm.ru/ |
|  | Sevastopol | Collection of Living Cultures of Plankton Microalgae | IBSS | 60 | WDCM |  |
|  | Sankt Petersburg | Collection for Plant Protection, All-Russian Institute of Plant Protection | VIZR | 785 | WDCM |  |
|  |  | Collection of Algae St. Petersburg (Leningrad) State University | CALU | 993 | WDCM | https://researchpark.spbu.ru/collection-ccem-rus/1628-ccem-kollekciya-calu-rus |
|  |  | Collection of Nonpathogenic Microorganisms for Agriculture | CIAM | 4781 | WDCM | https://arriam.spb.ru/ |
|  |  | Cryobank of Microorganisms-Destructors | LTI | 323 | WDCM |  |
|  |  | Culture Collection of Ciliates and Their Symbionts | CCCS | 1780 | WDCM |  |
|  |  | International Collection of Epidemic Strains | ICES | 2225 | WDCM |  |
|  |  | Komarov Botanical Institute Basidiomycetes Culture Collection | LE-BIN | 3882 | GCM, WFCC | https://vkm.ru/Cosolidated.htm |
|  |  | Peterhof Genetic Collection of Microalgae | PGC | 700 | WDCM |  |
|  |  | Resource Centre Culture Collection of Microorganisms | RC CCM | 548 | WDCM | https://researchpark.spbu.ru/en/ccem-eng |
|  |  | Russian Collection of Agricultural Microorganisms | RCAM | 5308 | WDCM | https://arriam.spb.ru/ |
|  | Syktyvkar | Strain Collection of Microalgae and Cyanobacteria from Northern and Arctic Regions in The Institute of Biology of Komi Scientific Centre | SYKOA | 498 | GCM, WDCM | https://ib.komisc.ru/sykoa/eng/collection |
|  | Ufa | Bashkortostan Collection of Algae and Cyanobacteria | BCAC | 1220 | WDCM, WFCC |  |
|  |  | Collection of Soil Microorganisms "Symbiont" | CSMS | 1000 | WDCM |  |
| Sweden | Gävleborg | University of Gävle Culture Collection | UGCC | 320 | WDCM |  |
|  | Göteborg | Culture Collection University of Goteborg | CCUG | 40500 | WDCM, WFCC, ECCO | https://ccug.se |
|  |  | Fungal Cultures University of Goteborg | FCUG | 9000 | WDCM |  |
|  |  | The Algal Bank | GUMACC | 80 |  | https://gu.se/en/marina-vetenskaper/about-us/algal-bank-gumacc |
|  | Kalmar | Kalmar Algae Collection | KAC | 56 |  | https://lnu.se/en/research/searchresearch/marine-phytoplankton-ecology-and-applications/ |
|  | Stockholm | Diatoms – the Kolbe Collection |  | 9000 |  | https://nrm.se/engelska/in-english/collections/palaeontological-collections---fossils |
|  | Uppsala | Uppsala University Culture Collection of Fungi | UPSC | 3200 | WDCM |  |

Microbial culture collections vary in size, the types of microorganisms maintained, institutional objectives, and formal status. Many microbial culture collections are affiliated with international organizations such as the World Federation for Culture Collections (WFCC), the World Data Centre for Microorganisms (WDCM), and the European Culture Collection Organisation (ECCO). In 2022, a European research infrastructure consortium dedicated to microbial resources was established, the Microbial Resource Research Infrastructure (MIRRI-ERIC) (https://www.mirri.org). MIRRI-ERIC provides access to expertise, services, and microbial strains from over 50 cultural collections and research institutes in several European countries, including Belgium, France, Greece, Latvia, Portugal and Spain.

**Table S4.** List of available Baltic bacteria genomes retrieved from Bac*Dive* (The Bacterial Diversity Database, https://bacdive.dsmz.de/).

| **Strain** | **Assembly level** | **BioSample** | **Assembly** | **Geographic Location** | **Isolation source** | **Collection date** | **Reference** |
| --- | --- | --- | --- | --- | --- | --- | --- |
| *Ahrensia kielensis* B9  (DSM 5890) | scaffold | SAMN02441016 | GCA_000374465 | Baltic Sea | brackish water | before 1990 | Uchino et al., 1998 |
| *Aquiflexum balticum* BA160 (DSM 16537) | chromosome | SAMN00777080/ | GCA_900176595 | Central Baltic Sea | brackish water | 1998 | Brettar et al., 2004a |
| *Arsukibacterium perlucidum* BA 131  (*Rheinheimera perlucida*) (DSM 18276) | scaffold | SAMN02441649 | GCA_000382165.1 | Central Baltic Sea | brackish water | 1998 | Brettar et al., 2006 |
| *Belliella baltica* BA134  (DSM 15883) | complete genome | SAMN02232009 | GCA_000265405.1 | Central Baltic Sea | brackish water | 1998 | Brettar et al., 2004b |
| *Blastococcus aggregatus* B15 (DSM 4725) | contig | SAMN05660748 | GCA_900221005.1 | Baltic Sea | brackish water | before 1988 | Urzi et al., 2004 |
| *Blastopirellula marina*  (DSM 3645) | scaffold | SAMN02436112 | GCA_000153105.1 | Kiel Fjord | brackish water | before 1986 | Schlesner et al., 2004 |
| *Cellulophaga baltica*  (DSM 24729) | scaffold | SAMN04487992 | GCA_900102165.1 | Bornholm | brown alga  *Fucus serratus* L. | 1995 | Johansen et al., 1999 |
| *Desulfosporosinus fructosivorans* 63.6F | scaffold | SAMN11254866 | GCA_004766045.1 | Little Belt | marine subsurface sediment | 2013 | Vandieken et al., 2017 |
| *Desulfosporosinus nitroreducens* 59.4F  (DSM 101562) | contig | SAMN28463191 | GCA_023897015.1 | Little Belt | marine subsurface sediment | 2013 | Vandieken et al., 2017 |
| *Erythrobacter sanguineus* A91 (DSM 11032) | contig | SAMN02745193 | GCA_900143235.1 | Baltic Sea | brackish water | before 1990 | Xu et al., 2020 |
| *Hirschia baltica* (DSM 5838) | complete genome | SAMN02598497 | GCA_000023785.1 | Kiel Fjord | brackish water | 1982 | Chertkov et al., 2011 |
| *Hoeflea marina* Ahrens  A43 (DSM 16791) | scaffold | SAMN09074944 | GCA_003182275.1 | Baltic Sea | brackish water | before 2004 | Peix et al., 2005 |
| *Idiomarina baltica*  OS145 (DSM 15154) | scaffold | SAMN02436083 | GCA_000152885.1 | Central Baltic Sea | brackish water | 1986 | Brettar et al., 2003 |
| *Kiloniella laminariae*  LD81 (DSM 19542) | scaffold | SAMN02441694 | GCA_000374005 | Kiel Bay | brown alga  *Laminaria saccharina* | 2003 | Wiese et al., 2009 |
| *Krasilnikoviella flava* (*Promicromonospora flava*) (DSM 21481) | scaffold | SAMN04324258 | GCA_900167525 | Kiel Bay | sediment | 2006 | Jiang et al., 2009 |
| *Labilibaculum filiforme* 59.16B (DSM 101180) | scaffold | SAMN06266691 | GCA_002843315.1 | Little Belt | marine subsurface sediment | 2013 | Vandieken et al., 2018 |
| *Labilibaculum manganireducens*  59.10-2M (DSM 102944) | contig | SAMN06266628 | GCA_002843385 | Little Belt | marine subsurface sediment | 2013 | Vandieken et al., 2018 |
| *Marinisporobacter balticus* 59.4M (DSM 102940) | contig | SAMN10864736 | GCA_004345705 | Little Belt | marine subsurface sediment | 2013 | Vandieken et al., 2018 |
| *Posidoniimonas polymericola* Pla123a (DSM 103020) | contig | SAMN10954022 | GCA_007859935.1 | Heiligendamm | wood incubated in brackish water | 2014 | Wiegand et al., 2020 |
| *Pseudorhodobacter ferrugineus* A7 (DSM 5888) | contig | SAMN02441236 | GCA_000420745.1 | Baltic Sea | brackish water | before 1990 | Uchino et al., 2002 |
| *Rheinheimera baltica* OSBAC1 (DSM 14885) | scaffold | SAMN02441194 | GCA_000425345.1 | Central Baltic Sea | brackish water | before 2002 | Brettar et al., 2002 |
| *Rhodopirellula baltica* SH 1 (DSM 10527) | complete genome | SAMEA3138332 | GCA_000196115.1 | Kiel Fjord | brackish water | before 1996 | Glöckner et al., 2003 |
| *Rhodopirellula heiligendammensis* Poly21 (DSM 102266) | scaffold | SAMN10954034 | GCA_007860105.1 | Heiligendamm | polyethylene particles incubated in brackish water | 2015 | Kallscheuer et al., 2020a |
| *Roseibium aggregatum* B1 (DSM 13394) | contig | SAMN02436089 | GCA_000168975.1 | Baltic Sea | sediment | before 2000 | Uchino et al., 1998 |
| *Rubripirellula tenax* Poly51 (DSM 103356) | scaffold | SAMN10954035 | GCA_007860125.1 | Heiligendamm | polyethylene particles incubated in brackish water | 2014 | Kallscheuer et al. 2020b |
| *Ruegeria gelatinovorans* B6 (*Thalassobius gelatinovorus*) (DSM 5887) | contig | SAMN04488043 | GCA_900111045.1 | Kiel Fjord | sediment | before 1990 | Arahal et al., 2005 |
| *Shewanella baltica* OS155 (DSM 16281) | complete genome | SAMN02598299 | GCA_000015845.1 | Central Baltic Sea | brackish water | 1986 | Ziemke et al., 1998 |
| *Shewanella denitrificans* OS217 (DSM 15013) | complete genome | SAMN02598300 | GCA_000013765.1 | Gotland Deep | brackish water | 1986 | Brettar et al., 2002 |
| *Shewanella glacialipiscicola* T147 | contig | SAMD00284632 | GCA_019655235.1 | Baltic Sea | cod | 1996 | Satomi et al., 2007 |
| *Stieleria varia* Pla52n  (DSM 102885) | scaffold | SAMN10954025 | GCA_007860045.1 | Heiligendamm | wood incubated in brackish water | 2014 | Surup et al., 2020 |
| *Sulfurimonas gotlandica* GD1 (DSM 19862) | complete genome | SAMN02470075 | GCA_000242915.2 | Gotland Deep | sulfidic water of a pelagic redox zone | 2005 | Labrenz et al., 2013 |

*Norway is included because, until 2016, its resources contained strains from the Scandinavian Culture Collection of Algae and Protozoa (SCCAP) at the University of Copenhagen (UoC), Denmark.

**Only the culture collection from the European part of Russia is included.

***These collections have the status of an International Depositary Authority (IDA) recognized by WIPO and accept microbial deposits under the Budapest Treaty on the International Recognition of the Deposit of Microorganisms for the Purposes of Patent Procedure.

(WDCM – the World Data Centre for Microorganisms; ECCO – the European Culture Collection Organisation; WFCC – the World Federation for Culture Collections; GCM – the Global Catalogue of Microorganism).

**Table S5.** List of available genomes from Cyanobacteriota/Melainabacteria group from the Baltic Sea, retrieved from NCBI genome database (https://ncbi.nlm.nih.gov/datasets/genome/#!/prokaryotes/).

| **Strain** | **Assembly level** | **BioSample** | **Assembly** | **Geographic Location** | **Isolation source** | **Collection date** | **Sample type** | **Reference** |
| --- | --- | --- | --- | --- | --- | --- | --- | --- |
| *Anabaena* sp. UHCC 0187 | scaffold | SAMN12084330 | GCA_009712035.1 | Porkkala | brackish water | 1999 | Cell culture | Österholm et al., 2020 |
| *Anabaena* sp. UHCC 0204 | scaffold | SAMN12084250 | GCA_009711975.1 | Porkkala | brackish water | 1999 | Cell culture | Österholm et al., 2020 |
| *Anabaena* sp. UHCC 0253 | scaffold | SAMN12084370 | GCA_009712085.1 | Porkkala | brackish water, epiphytic | 1999 | Cell culture | Österholm et al., 2020 |
| *Aphanizomenon flos-aquae* KM1D3_PB | complete | SAMN14448365 | GCA_017346875.1 | Curonian Lagoon | brackish water | 2012 | Unialgal non-axenic culture | Dreher et al., 2021;  Šulčius et al., 2015 |
| *Aphanizomenon* sp. UHCC 0183 | scaffold | SAMN12084337 | GCA_009712065.1 | Finland | brackish water | 1993 | Cell culture | Österholm et al., 2020 |
| *Cyanobium usitatum* str. Tous | complete | SAMEA115019959 | GCA_963920485.1 | Baltic Sea Proper | brackish water | 2020 | Cell culture | Aguilera et al., 2023 |
| *Dolichospermum* sp. UHCC 0259 | scaffold | SAMN12084251 | GCA_009711985.1 | Gulf of Finland | brackish water | 2004 | Cell culture | Österholm et al., 2020 |
| *Dolichospermum* sp. UHCC 0260 | scaffold | SAMN12084254 | GCA_009711965.1 | Gulf of Finland | brackish water | 2004 | Cell culture | Österholm et al., 2020 |
| *Dolichospermum* sp. UHCC 0315A | complete | SAMN05967626 | GCA_008121535.1 | Gulf of Finland | brackish water | 2015 | DNA sample | Teikari et al., 2018 |
| *Nodularia sphaerocarpa* UHCC 0038 | complete | SAMN15769246 | GCA_022376295.1 | Baltic Sea | brackish water | 1978 | Cell culture | Ahmed et al., 2021 |
| *Nodularia spumigena* CCY9414 | chromosome | SAMN01915661 | GCA_000340565.3 | Bornholm | brackish water | 1994 | Cell culture | Voss et al., 2013 |
| *Nodularia spumigena*  UHCC 0039 | complete | SAMN05967630 | GCA_003054475.1 | Gulf of Finland | brackish water | 2015 | DNA sample | Teikari et al., 2018 |
| *Nostoc edaphicum* CCNP1411 | complete | SAMN15196599 | GCA_014023275.1 | Gulf of Gdańsk | brackish water | 2010 | Cell culture | Fidor et al., 2020 |
| *Nostoc* sp.  UHCC 0870 | complete | SAMN25514200 | GCA_022063185.1 | Varlaxudden | brackish water | 2014 | Cell culture | Heinilä et al., 2022 |
| *Pseudanabaena galeata*  CCNP1313 | complete | SAMN31016687 | GCA_029910235.1 | Gulf of Gdańsk | brackish water | 2010 | Cell culture | Grabski et al., 2024 |
| *Synechococcus* sp. CBW1107  isolate KAC_102 | complete | SAMEA115019960 | GCA_963920495.1 | Baltic Sea | brackish water | 2023 | Cell culture | Aguilera et al., 2023 |
| *Synechococcus* sp. CBW1107  isolate KAC_105 | complete | SAMEA115019958 | GCA_963920475.1 | Baltic Sea | brackish water | 2020 | Cell culture | Aguilera et al., 2023 |
| *Synechococcus* sp. CBW1107  isolate KAC_106 | complete | SAMEA115019961 | GCA_963920505.1 | Baltic Sea | brackish water | 2023 | Cell culture | Aguilera et al., 2023 |
| *Synechococcus* sp. CBW1107  isolate KAC_114 | complete | SAMEA115019962 | GCA_963920465.1 | Baltic Sea | brackish water | 2020 | Cell culture | Aguilera et al., 2023 |
| Uncultured cyanobacterium (MAG*) | complete | SAMEA114813976 | GCA_963702855.1 | Zealand, harbour | saltwater | 2022 | Marine metagenome | NP** |
| uncultured cyanobacterium (MAG*) | complete | SAMEA114813212 | GCA_963703315.1 | Denmark | sediment | 2020 | Sediment metagenome | NP** |
| Uncultured *Cyanobium* sp. (MAG*) | scaffold | SAMEA117384603 | GCA_964621985.1 | Easter Gotland Basin | brackish water | 2018 | Aquatic metagenome | NP** |
| *Waterburya agarophytonicola*  KI4 | scaffold | SAMN16869237 | GCA_020640915.1 | Germany | symbiont of *Gracilaria* *vermiculophylla* | 2017 | mixed culture | Bonthond et al., 2021 |

**Table S6.** Bacteriophages from Baltic Sea with sequenced genomes available in NCBI Database.

| **Phage name** | **Bacterial host** | **ICTV*** | **Genome size (bp)** | **GenBank accession number** | **Geographic location and sample type** | **Collection date** | **Reference** |
| --- | --- | --- | --- | --- | --- | --- | --- |
| Cellulophaga phage φ39:1 | *Cellulophaga baltica* | UC | 28 760 | NC_021804 | Øresund; surface water | 2000 | Holmfeldt et al., 2007; Šulčius and Holmfeldt, 2016 |
| Cellulophaga phage φ46:1 | *Cellulophaga baltica* | UC | 34 844 | NC_021800 |  | 2000 |  |
| Cellulophaga phage φST | *Cellulophaga baltica* | *Cbastvirus* | 79 114 | NC_020842 |  | 2000 |  |
| Cellulophaga phage φ19:2 | *Cellulophaga baltica* | *Cbastvirus* | 78 275 | KC821621 |  | 2000 |  |
| Cellulophaga phage φ13:1 | *Cellulophaga baltica* | *Cbastvirus* | 76 666 | KC821625 |  | 2000 |  |
| Cellulophaga phage φ10:1 | *Cellulophaga baltica* | *Cebadecemvirus* | 53 664 | NC_021802 |  | 2000 |  |
| Cellulophaga phage φ19:1 | *Cellulophaga baltica* | *Cellubavirus* | 57 447 | NC_021799 |  | 2000 |  |
| Cellulophaga phage φ12:1 | *Cellulophaga baltica* | *Helsingorvirus* | 39 148 | NC_021791 |  | 2000 |  |
| Cellulophaga phage φ12:3 | *Cellulophaga baltica* | *Helsingorvirus* | 39 151 | KC821615 |  | 2000 |  |
| Cellulophaga phage φ17:1 | *Cellulophaga baltica* | *Helsingorvirus* | 38 776 | NC_021795 |  | 2000 |  |
| Cellulophaga phage φ18:1 | *Cellulophaga baltica* | *Helsingorvirus* | 39 189 | NC_021790 |  | 2000 |  |
| Cellulophaga phage φ18:2 | *Cellulophaga baltica* | *Helsingorvirus* | 38 476 | KC821627 |  | 2000 |  |
| Cellulophaga phage φSM | *Cellulophaga baltica* | UC | 54 | NC_020860 |  | 2000 |  |
| Cellulophaga phage φ3:1 | *Cellulophaga baltica* | UC | 54 427 | KC821630 |  | 2000 |  |
| Cellulophaga phage φ3ST:2 | *Cellulophaga baltica* | UC | 54 015 | KC821610 |  | 2005 |  |
| Cellulophaga phage φ38:2 | *Cellulophaga baltica* | UC | 54 012 | KC821629 |  | 2005 |  |
| Cellulophaga phage φ47:1 | *Cellulophaga baltica* | UC | 54 016 | KC821634 |  | 2005 |  |
| Cellulophaga phage φ48:2 | *Cellulophaga baltica* | UC | 11 703 | NC_021793 |  | 2005 |  |
| Cellulophaga phage φ14:2 | *Cellulophaga baltica* | *Akihdevirus* | 100 418 | NC_021806 |  | 2005 |  |
| Cellulophaga phage φ46:3 | *Cellulophaga baltica* | *Bacelvirus* | 72 961 | NC_021792 |  | 2005 |  |
| Cellulophaga phage φ13:2 | *Cellulophaga baltica* | *Baltivirus* | 72 369 | NC_021803 |  | 2005 |  |
| Cellulophaga phage φ18:3 | *Cellulophaga baltica* | *Baltivirus* | 71 443 | NC_021794 |  | 2005 |  |
| Cellulophaga phage φ19:3 | *Cellulophaga baltica* | *Baltivirus* | 75 994 | NC_021789 |  | 2005 |  |
| Cellulophaga phage φ38:1 | *Cellulophaga baltica* | *Callevirus* | 72 534 | NC_021796 |  | 2005 |  |
| Cellulophaga phage φ40:1 | *Cellulophaga baltica* | *Callevirus* | 72 529 | KC821612 |  | 2005 |  |
| Cellulophaga phage φ4:1 | *Cellulophaga baltica* | *Lightbulbvirus* | 145 865 | NC_021788 |  | 2005 |  |
| Cellulophaga phage φ17:2 | *Cellulophaga baltica* | *Lightbulbvirus* | 145 343 | NC_021798 |  | 2005 |  |
| Cellulophaga phage φ12:2 | *Cellulophaga baltica* | *Microviridae* | 6 453 | NC_021797 |  | 2005 |  |
| Cellulophaga phage φ12a:1 | *Cellulophaga baltica* | *Microviridae* | 6 478 | NC_021805 |  | 2005 |  |
| Cellulophaga phage φ18:4 | *Cellulophaga baltica* | *Microviridae* | 6 478 | KC821628 |  | 2005 |  |
| Cellulophaga phage φ48:1 | *Cellulophaga baltica* | *Microviridae* | 6 478 | KC821631 |  | 2005 |  |
| Shewanella sp. phage 1/4 | *Shewanella frigidimarina* | UC | 133 824 | NC_025436 |  | 2011 |  |
| Shewanella sp. phage 1/40 | *Shewanella baltica* | UC | 139 004 | NC_025470 | the Hanko Peninsula; | 2011 | Luhtanen et al., 2014; |
| Shewanella sp. phage 1/41 | *Shewanella baltica* | UC | 43 540 | NC_025458 | sea ice samples | 2011 | Senčilo et al., 2015 |
| Shewanella sp. phage 1/44 | *Shewanella frigidimarina* | UC | 49 640 | NC_025463 |  | 2011 |  |
| Shewanella sp. phage 3/49 | *Shewanella baltica* | UC | 40 161 | NC_025466 |  | 2011 |  |
| Flavobacterium sp. phage 1/32 | *Flavobacterium sp. gelidilacus* | UC | 42 252 | KJ018210 |  | 2011 |  |
| Cyanophage vB_AphaS-CL131 | *Aphanizomenon flos-aquae* | UC | 112 793 | MG209611 | Curonian Lagoon; brackish water | 2012 | Šulčius et al., 2015;  Šulčius et al., 2019 |
| Rheinheimera phage vB_RspM-Barba1A - Rheinheimera phage vB_RspM-Barba35A (54 phages)*** | *Rheinheimera* sp. | *Barbavirus* | 80 203-84 615 | MK719701-MK719754 | Linnaeus Microbial Observatory (LMO) station (Sweden); surface water | 2015 | Nilsson et al., 2019 |
|  |  |  |  |  |  |  |  |
| Flavobacterium phage vB_FspS_hattifnatt9-1 | *Flavobacterium* sp. | *Hattifnattvirus* | 39 262 | MN812207 | Linnaeus Microbial Observatory (LMO) station (Sweden); surface water | 2015 | Nilsson et al., 2020 |
| Flavobacterium phage vB_FspS_laban6-1 | *Flavobacterium* sp. | *Labanvirus* | 43 236 | MN812211 |  | 2015 |  |
| Flavobacterium phage vB_FspS_morran9-1 | *Flavobacterium* sp. | *Lillamyvirus* | 38 385 | MN812219 |  | 2015 |  |
| Flavobacterium phage vB_FspS_hemulen6-1, 6-2, 9-1 (3 phages)*** | *Flavobacterium* sp. | *Lillamyvirus* | 38 901-39 030 | MN812208- MN812210 |  | 2015 |  |
| Flavobacterium phage vB_FspS_lillamy9-1- Flavobacterium phage vB_FspS_lillamy9-7  (7 phages)*** | *Flavobacterium* sp. | *Lillamyvirus* | 37 531-38 268 | MN812212- MN812218 |  | 2015 |  |
| Flavobacterium phage vB_FspS_sniff9-1 | *Flavobacterium* sp. | *Lillamyvirus* | 38 134 | MN812229 |  | 2015 |  |
| Flavobacterium phage vB_FspS_sniff9-2 | *Flavobacterium* sp. | *Lillamyvirus* | 37 907 | MN812230 |  | 2015 |  |
| Flavobacterium phage vB_FspS_stinky9-1 | *Flavobacterium* sp. | *Lillamyvirus* | 38 502 | MN812238 |  | 2015 |  |
| Flavobacterium phage vB_FspS_snork6-1, 6-2, 9-1 (3 phages)*** | *Flavobacterium* sp. | *Lillamyvirus* | 37 732-38 214 | MN812231- MN812233 |  | 2015 |  |
| Flavobacterium phage vB_FspS_tooticki6-1 | *Flavobacterium* sp. | *Muminvirus* | 36 912 | MN812240 |  | 2015 |  |
| Flavobacterium phage vB_FspS_tooticki9-1 | *Flavobacterium* sp. | *Muminvirus* | 36 912 | MN812241 |  | 2015 |  |
| Flavobacterium phage vB_FspS_filifjonk9-1 | *Flavobacterium* sp. | *Muminvirus* | 38 028 | MN812206 |  | 2015 |  |
| Flavobacterium phage vB_FspS_mymlan6-1 | *Flavobacterium* sp. | *Muminvirus* | 38 501 | MN812227 |  | 2015 |  |
| Flavobacterium phage vB_FspS_mymlan6-2 | *Flavobacterium* sp. | *Muminvirus* | 38 372 | MN812228 |  | 2015 |  |
| Flavobacterium phage vB_FspS_snusmum6-1,6-2,6-3,9-1 (4 phages)*** | *Flavobacterium* sp. | *Muminvirus* | 38 743-39 365 | MN812234-MN812237 |  | 2015 |  |
| Flavobacterium phage vB_FspS_mumin6-1, 6-2, 6-3, 6-4 and Flavobacterium phage vB_FspS_mumin9-1, 9-2, 9-3 (7 phages)*** | *Flavobacterium* sp. | *Muminvirus* | 37 532-38 527 | MN812220- MN812226 |  | 2015 |  |
| Flavobacterium phage vB_FspM_pippi8-1 | *Flavobacterium* sp. | *Pippivirus* | 34 711 | MN812205 |  | 2015 |  |
| Flavobacterium phage vB_FspM_lotta8-1 | *Flavobacterium* sp. | *Pippivirus* | 34 751 | MN812203 |  | 2015 |  |
| Flavobacterium phage vB_FspM_lotta8-2 | *Flavobacterium* sp. | *Pippivirus* | 34 627 | MN812204 |  | 2015 |  |
| Flavobacterium phage vB_FspS_tant8-1 | *Flavobacterium* sp. | *Tantvirus* | 40 542 | MN812239 |  | 2015 |  |
| Flavobacterium phage vB_FspP_elemoA_1-5B - Flavobacterium phage vB_FspP_elemoA_15-9C (52 phages)*** | *Flavobacterium* sp. | *Elemovirus* | 60 061-61 691 | MN497017-MN497125** | Linnaeus Microbial Observatory (LMO) station (Sweden); surface water | 2016 | Hoetzinger et al., 2021 |
| Flavobacterium phage vB_FspP_elemoB_14-3B | *Flavobacterium* sp. | *Elemovirus* | 60 925 | MT497068 |  | 2016 |  |
| Flavobacterium phage vB_FspP_elemoC_13-1C | *Flavobacterium* sp. | *Elemovirus* | 60 458 | MT497066 |  | 2016 |  |
| Flavobacterium phage vB_FspP_elemoC_14-1A | *Flavobacterium* sp. | *Elemovirus* | 61 048 | MT497067 |  | 2016 |  |
| Flavobacterium phage vB_FspP_elemoC_15-9A | *Flavobacterium* sp. | *Elemovirus* | 60 162 | MT497098 |  | 2016 |  |
| Flavobacterium phage vB_FspP_elemoC_3-9B | *Flavobacterium* sp. | *Elemovirus* | 60 198 | MT497107 |  | 2016 |  |
| Flavobacterium phage vB_FspP_elemoD_13-5B | *Flavobacterium* sp. | *Elemovirus* | 60 393 | MT497091 |  | 2016 |  |
| Flavobacterium phage vB_FspP_elemoE_6-9C | *Flavobacterium* sp. | *Elemovirus* | 59 979 | MT497072 |  | 2016 |  |
| Flavobacterium phage vB_FspP_elemoE_10-3D | *Flavobacterium* sp. | *Elemovirus* | 59 743 | MT497078 |  | 2016 |  |
| Flavobacterium phage vB_FspP_elemoF_6-3D | *Flavobacterium* sp. | *Elemovirus* | 59 846 | MT497071 |  | 2016 |  |
| Flavobacterium phage vB_FspM_immuto_2-6A | *Flavobacterium* sp. | *Immutovirus* | 160 410 | MW353175 |  | 2016 |  |
| Flavobacterium phage vB_FspM_immuto_3-5A | *Flavobacterium* sp. | *Immutovirus* | 160 366 | MW353176 |  | 2016 |  |
| Flavobacterium phage vB_FspM_immuto_13-6C | *Flavobacterium* sp. | *Immutovirus* | 160 366 | MW353177 |  | 2016 |  |
| Staphylococcus phage BSwM-KMM1 | *Staphylococcus* sp. | UC | 137 386 | OP902294.1 | the Kiel fjord; surface water | 2021 | Stante et al., 2024 |
| Citrobacter phage BSwM KMM2 | *Citrobacter freundii* | *Mooglevirus* | 88 537 | OP902295.1 |  | 2021 |  |
| Citrobacter phage BSwS KMM4 | *Citrobacter* sp. | *Mooglevirus* | 86 911 | OP902293.1 |  | 2021 |  |
| Citrobacter phage BSwS KMM3 | *Citrobacter* sp. | *Tlsvirus* | 49 164 | OP902292.1 |  | 2021 |  |

**Table S7**. Overview of access and benefit sharing policy in nine Baltic coastal countries.

| Country | Nagoya Status | Competent National Authority | ABS National Focal Point | Access rules |
| --- | --- | --- | --- | --- |
| Denmark | since  12.10.2014 | Danish Environmental Protection Agency | established | no access requirements are in place |
| Estonia | since 19.03.2019 | Ministry of the Environment (wildlife)  Ministry of Rural Affairs (agriculture) | established | no access requirements are in place |
| Finland | since 01.09.2016 | Finnish Environment Institute | established | no access requirements for GR are in place; however, access to traditional knowledge of indigenous and local communities is regulated |
| Germany | since 20.07.2016 | Federal Agency for Nature Conservation | established | no access requirements are in place |
| Latvia | non-party | no data in ABS portal  (The Ministry of Environmental Protection and Regional Development  National*  Botanical Garden for tasks stated in part 7 of the EU ABS regulations)* | established | no data in ABS portal |
| Lithuania | non-party | no data in ABS portal  (Ministry of Environment)* | established | no data in ABS portal |
| Poland | non-party | Ministry of the Environment | established | no access requirements are in place |
| Sweden | since 07.12.2016 | The Swedish Environmental Protection Agency | established | no access requirements are in place |
| Russia | non-party | no data in ABS portal | no data in ABS portal | no data in ABS portal |

* Information based on reports sent by CNA to the EC, accessed on June 6^th^ 2023, via EU CIRCABC platform for information exchange at https://circabc.europa.eu/ui/group/3f466d71-92a7-49eb-9c63-6cb0fadf29dc/library/ddb37bea-f550-4504-ad31-3375dce1f37e.

**References:**

1. Accelerating Algae Product Developments in Baltic and North Sea Project website. https://cordis.europa.eu/project/id/101112943 (accessed 28 February 2025).
2. Aguilera, A., Alegria Zufia, J., Bas Conn, L., Gurlit, L., Śliwińska-Wilczewska, S., Budzałek, G. (2023). Ecophysiological analysis reveals distinct environmental preferences in closely related Baltic Sea picocyanobacteria. *Environ Microbiol.* 25(9), 1674–1695. https://doi.org/10.1111/1462-2920.16384.
3. Ahmed, M.N., Wahlsten, M., Jokela, J., Nees, M., Stenman, U.H., Alvarenga, D.O., et al. (2021). Potent inhibitor of human trypsins from the aeruginosin family of natural products. *ACS Chem Biol.* 16(11), 2537–2546. https://doi.org/10.1021/acschembio.1c00611.
4. All-Russian Collection of Microorganisms. https://vkm.ru/index.htm (accessed 28 February 2025).
5. Arahal, D.R., Macián, M.C., Garay, E., Pujalte, M.J. (2005). *Thalassobius mediterraneus* gen. nov., sp. nov., and reclassification of *Ruegeria gelatinovorans* as *Thalassobius gelatinovorus* comb. nov. *Int J Syst Evol Microbiol.* 55, 2371–2376. https://doi.org/10.1099/ijs.0.63842-0.
6. BioCC Microbial Collection. https://eccosite.org/portfolio-item/biocc-mc/ (accessed 28 February 2025).
7. Biotrino APS website. https://biotrino.com (accessed 28 February 2025).
8. Biovento website. https://biovento.wixsite.com/biovento (accessed 28 February 2025).
9. Bonthond, G., Shalygin, S., Bayer, T., Weinberger, F. (2021). Draft genome and description of *Waterburya agarophytonicola* gen. nov. sp. nov. (Pleurocapsales, Cyanobacteria): a seaweed symbiont. *Antonie Van Leeuwenhoek.* 114(12), 2189–2203. https://doi.org/10.1007/s10482-021-01672-x.
10. Brettar, I., Christen, R., Höfle, M.G. (2002). *Shewanella denitrificans* sp. nov., a vigorously denitrifying bacterium isolated from the oxic-anoxic interface of the Gotland Deep in the central Baltic Sea. *Int J Syst Evol Microbiol.* 52, 2211–2217. http://www.ncbi.nlm.nih.gov/pubmed/12508890.
11. Brettar, I., Christen, R., Höfle, M.G. (2003). *Idiomarina baltica* sp. nov., a marine bacterium with a high optimum growth temperature isolated from surface water of the central Baltic Sea. *Int J Syst Evol Microbiol*. 53(2), 407–413. https://doi.org/10.1099/ijs.0.02399-0.
12. Brettar, I., Christen, R., Höfle, M.G. (2004a). *Aquiflexum balticum* gen. nov., sp. nov., a novel marine bacterium of the *Cytophaga*-*Flavobacterium*-*Bacteroides* group isolated from surface water of the central Baltic Sea. *Int J Syst Evol Microbiol*. 54(6), 2335–2341. https://doi.org/10.1099/ijs.0.63255-0.
13. Brettar, I., Christen, R., Höfle, M.G. (2004b). *Belliella baltica* gen. nov., sp. nov., a novel marine bacterium of the *Cytophaga*-*Flavobacterium*-*Bacteroides* group isolated from surface water of the central Baltic Sea. *Int J Syst Evol Microbiol.* 54(1), 65–70. https://doi.org/10.1099/ijs.0.02752-0.
14. Brettar, I., Christen, R., Höfle, M.G. (2006). *Rheinheimera perlucida* sp. nov., a marine bacterium of the Gammaproteobacteria isolated from surface water of the central Baltic Sea. *Int J Syst Evol Microbiol*. 56(9), 2177–2183. https://doi.org/10.1099/ijs.0.64172-0.
15. CCCryo – Culture Collection of Cryophilic Algae. https://cccryo.fraunhofer.de/ (accessed 28 February 2025).
16. Central Collection of Algal Cultures. https://uni-due.de/biology/ccac/ (accessed 28 February 2025).
17. Chertkov, O., Brown, P.J., Kysela, D.T., de Pedro, M.A., Lucas, S., Copeland, A., et al. (2011). Complete genome sequence of *Hirschia baltica* type strain (IFAM 1418(T)). *Stand Genomic Sci*. 5(3), 287–297. https://doi.org/10.4056/sigs.2205004.
18. Collection of Algae St. Petersburg (Leningrad) State University. https://researchpark.spbu.ru/collection-ccem-rus/1628-ccem-kollekciya-calu-rus (accessed 28 February 2025).
19. Collection of Environmental and Laboratory Microbial Strains. https://https://eemb.ut.ee/eng/celms_english_introduction_list.php (accessed 28 February 2025).
20. Collection of Eubiotic and Epiphytic Microorganisms. https://mb.kubsau.ru/ (accessed 28 February 2025).
21. Collection of Industrial Microorganisms. https://cim.ibprs.pl/ (accessed 28 February 2025).
22. Collection of Nonpathogenic Microorganisms for Agriculture. https://arriam.spb.ru/.
23. Collection of Plasmids and Microorganisms. https://kpd.ug.edu.pl (accessed 28 February 2025).
24. Collection of Reproductive Tract Microorganisms. https://https://eemb.ut.ee/humb_eesti_Contacts_list.php (accessed 28 February 2025).
25. Collection of Rhizosphere Microorganisms. https://collection.ibppm.ru/ (accessed 28 February 2025).
26. Culture Collection of Algae at Göttingen University. https://epsag.uni-goettingen.de/ (accessed 28 February 2025).
27. Culture Collection of Baltic Algae. https://ccba.ug.edu.pl (accessed 28 February 2025).
28. Culture Collection of Microalgae and Cyanobacteria IPPAS. https://cellreg.org/Catalog/ (accessed 28 February 2025).
29. Culture Collection of Microorganism IO PAN. https://old.iopan.pl/BioChem/collections.html (accessed 28 February 2025).
30. Culture Collection of Wild and Cultivated Macromycetes. https://mycol-algol.ru/collection_2.html (accessed 28 February 2025).
31. Culture Collection University of Goteborg. https://ccug.se (accessed 28 February 2025).
32. Diatoms – the Kolbe Collection. https://nrm.se/engelska/in-english/collections/palaeontological-collections---fossils (accessed 28 February 2025).
33. Dreher, T.W., Davis, E.W. 2nd, Mueller, R.S. (2021). Complete genomes derived by directly sequencing freshwater bloom populations emphasize the significance of the genus level ADA clade within the Nostocales. *Harmful Algae.* 103, 102005. https://doi.org/10.1016/j.hal.2021.102005.
34. DSMZ – German Collection of Microorganisms and Cell Cultures GmbH. https://dsmz.de (accessed 28 February 2025).
35. Ekogina website. https://powdi.lt (accessed 28 February 2025).
36. Exploring Marine Resources for Bioactive Compounds: From Discovery to Sustainable Production and Industrial Applications Project website. https://cordis.europa.eu/project/id/245137 (accessed 28 February 2025).
37. Fidor, A., Grabski, M., Gawor, J., Gromadka, R., Węgrzyn, G., Mazur-Marzec, H. (2020). *Nostoc edaphicum* CCNP1411 from the Baltic Sea - a new producer of nostocyclopeptides. *Mar Drugs.* 18(9), 442. https://doi.org/10.3390/md18090442.
38. FUME – Functional Metagenomics - Harnessing the Biotechnological Potential of Completely Novel Protein Families Project website. https://cordis.europa.eu/project/id/281384 (accessed 28 February 2025).
39. Furcella OÜ website. https://berrichi.eu (accessed 28 February 2025).
40. Glöckner, F.O., Kube, M., Bauer, M., Teeling, H., Lombardot, T., Ludwig, W., et al. (2003). Complete genome sequence of the marine planctomycete *Pirellula* sp. strain 1. *Proc Natl Acad Sci USA*. 100(14), 8298–8303. https://doi.org/10.1073/pnas.1431443100.
41. Grabski, M., Gawor, J., Cegłowska, M., Gromadka, R., Mazur-Marzec, H., Węgrzyn, G. (2024). Genome mining of *Pseudanabaena galeata* CCNP1313 indicates a new scope in the search for antiproliferative and antiviral agents. *Microorganisms*. 12(8), 1628. https://doi.org/10.3390/microorganisms12081628.
42. Hambi Culture Collection. https://helsinki.fi/en/infrastructures/biodiversity-collections/infrastructures/microbial-domain-biological-resource-centre-hambi (accessed 28 February 2025).
43. Heinilä, L.M.P., Jokela, J., Ahmed, M.N., Wahlsten, M., Kumar, S., Hrouzek, P., et al. (2022). Discovery of varlaxins, new aeruginosin-type inhibitors of human trypsins. *Org Biomol Chem.* 20(13), 2681–2692. https://doi.org/10.1039/d1ob02454j.
44. Herbarium Pomeranicum. https://herbariumpomeranicum.pl (accessed 28 February 2025).
45. Hoetzinger, M., Nilsson, E., Arabi, R., Osbeck, C.M.G., Pontiller, B., Hutinet, G., et al. (2021). Dynamics of Baltic Sea phages driven by environmental changes. *Environ Microbiol.* 23(8), 4576–4594. https://doi.org/10.1111/1462-2920.15651
46. Holmfeldt, K., Middelboe, M., Nybroe, O., Riemann, L. (2007). Large variabilities in host strain susceptibility and phage host range govern interactions between lytic marine phages and their *Flavobacterium* hosts. *Appl Environ Microbiol.* 73(21), 6730–6799. https://doi.org/10.1128/AEM.01399-07.
47. Hustedt Diatom Collection. https://awi.de/en/science/biosciences/polar-biological-oceanography/main-research-focus/hustedt-diatom-study-centre/diatom-collection.html (accessed 28 February 2025).
48. IBT Fungal Culture Collection. https://bioengineering.dtu.dk/research/strain-collections/ibt-culture-collection (accessed 28 February 2025).
49. InnoAerogel: Sustainable Sorbent for Aquatic Oil Spills Clean-Up Project website. https://cordis.europa.eu/project/id/101188620 (accessed 28 February 2025).
50. IOMB Strain Collection IO PAN. https://old.iopan.pl/MolBio/IOMB.html (accessed 28 February 2025).
51. Jena Microbial Resource Collection. https://leibniz-hki.de/de/jena-microbial-resource-collection.html (accessed 28 February 2025).
52. Jiang, Y., Wiese, J., Cao, Y.-R., Xu, L.-H., Imhoff, J.F., Jiang, C.-L. (2009). *Promicromonospora flava* sp. nov., isolated from sediment of the Baltic Sea. *Int J Syst Evol Microbiol*. 59, 1599–1602. https://doi.org/10.1099/ijs.0.006197-0.
53. Johansen, J.E., Nielsen, P., Sjøholm, C. (1999). Description of *Cellulophaga baltica* gen. nov., sp. nov. and *Cellulophaga fucicola* gen. nov., sp. nov. and reclassification of [Cytophaga] lytica to *Cellulophaga lytica* gen. nov., comb. nov. *Int J Syst Bacteriol.* 49(3), 1231–1240. doi: https://doi.org/10.1099/00207713-49-3-1231.
54. Kallscheuer, N., Jogler, M., Wiegand, S., Peeters, S.H., Heuer, A., Boedeker, C. (2020b). Three novel *Rubripirellula* species isolated from plastic particles submerged in the Baltic Sea and the estuary of the river Warnow in northern Germany. *Antonie Van Leeuwenhoek.* 113(12), 1767–1778. https://doi.org/10.1007/s10482-019-01368-3.
55. Kallscheuer, N., Wiegand, S., Jogler, M., Boedeker, C., Peeters, S.H., Rast, P., et al. (2020a). *Rhodopirellula heiligendammensis* sp. nov., *Rhodopirellula pilleata* sp. nov., and *Rhodopirellula solitaria* sp. nov. isolated from natural or artificial marine surfaces in Northern Germany and California, USA, and emended description of the genus *Rhodopirellula*. *Antonie Van Leeuwenhoek.* 113(12), 1737–1750. https://doi.org/10.1007/s10482-019-01366-5.
56. Kalmar Algae Collection. https://lnu.se/en/research/searchresearch/marine-phytoplankton-ecology-and-applications/ (accessed 28 February 2025).
57. Komarov Botanical Institute Basidiomycetes Culture Collection. https://vkm.ru/Cosolidated.htm (accessed 28 February 2025).
58. Labrenz, M., Grote, J., Mammitzsch, K., Boschker, H.T.S., Laue, M., Jost, G., et al. (2013). *Sulfurimonas gotlandica* sp. nov., a chemoautotrophic and psychrotolerant epsilonproteobacterium isolated from a pelagic redoxcline, and an emended description of the genus Sulfurimonas. *Int J Syst Evol Microbiol.* 63(11), 4141–4148. https://doi.org/10.1099/ijs.0.048827-0.
59. LOCALITY – Nature-Positive Algae-Based Food, Agriculture, Aquaculture and Textile Products Made in North and Baltic Sea Ecosystems Project website. https://cordis.europa.eu/project/id/101112884 (accessed 28 February 2025).
60. Luhtanen, A.-M., Eronen-Rasimus, E., Kaartokallio, H., Rintala, J.-M., Autio, R., Roine, E. (2014). Isolation and characterization of phage-host systems from the Baltic Sea ice. *Extremophiles.* 18(1), 121–130. https://doi.org/10.1007/s00792-013-0604-y.
61. Marine Biodiversity as Sustainable Resource of Disease-Suppressive Microbes and Bioprotectants for Aquaculture and Crop Diseases Project website. https://cordis.europa.eu/project/id/101000392 (accessed 28 February 2025).
62. Marine Microbial Biodiversity, Bioinformatics, Biotechnology Project website. https://cordis.europa.eu/project/id/287589 (accessed 28 February 2025).
63. Marine Microorganisms: Cultivation Methods for Improving their Biotechnological Applications Project website. https://cordis.europa.eu/project/id/311975 (accessed 28 February 2025).
64. Marine Research Laboratory at Syke Culture Collection. https://finmari-infrastructure.fi/facilities/laboratories/syke-mrc-marine-ecology-laborato/ (accessed 28 February 2025).
65. Microalgae and Zygnematophyceae Collection Hamburg. https://mzch-svck.uni-hamburg.de/ (accessed 28 February 2025).
66. Microbial Resource Research Infrastructure (MIRRI-ERIC). https://www.mirri.org.Wiese, J., Thiel, V., Nagel, K., Staufenberger, T., Imhoff, J.F. (2009). Diversity of antibiotic-active bacteria associated with the brown alga *Laminaria saccharina* from the Baltic Sea. *Mar Biotechnol (NY)*. 11(2), 287–300. https://doi.org/10.1007/s10126-008-9143-4.
67. Microbial Strain Collection of Latvia. https://mikro.daba.lv (accessed 28 February 2025).
68. National Center for Biotechnology Information. https://ncbi.nlm.nih.gov/datasets/genome/#!/prokaryotes/ (accessed 28 February 2025).
69. Natural History of Denmark Collections. https://samlinger.snm.ku.dk/en/dry-and-wet-collections/botany/algal-herbarium (accessed 28 February 2025).
70. Natural Products from Marine Fungi for the Treatment of Cancer Project website. https://cordis.europa.eu/project/id/265926 (accessed 28 February 2025).
71. New Biotechnological Approaches for Biodegrading and Promoting the Environmental Biotransformation of Synthetic Polymeric Materials Project website. https://cordis.europa.eu/project/id/312100 (accessed 28 February 2025).
72. Nilsson, E., Bayfield, O.W., Lundin, D., Antson, A.A., Holmfeldt, K. (2020). Diversity and host interactions among virulent and temperate Baltic Sea *Flavobacterium* Phages. *Viruses*. 12(2), 158. https://doi.org/10.3390/v12020158.
73. Nilsson, E., Li, K., Fridlund, J., Šulčius, S., Bunse, C., Karlsson, C.M.G., et al. (2019). Genomic and seasonal variations among aquatic phages infecting the Baltic Sea *Gammaproteobacterium* *Rheinheimera* sp. Strain BAL341. *App Environ Microbiol*. 85(18), e01003-19. https://doi.org/10.1128/AEM.01003-19.
74. Österholm, J., Popin, R.V., Fewer, D.P., Sivonen, K. (2020). Phylogenomic analysis of secondary metabolism in the toxic cyanobacterial genera *Anabaena*, *Dolichospermum* and *Aphanizomenon*. *Toxins (Basel).* 12(4), 248. https://doi.org/10.3390/toxins12040248.
75. Peix, A., Rivas, R., Trujillo, M.E., Vancanneyt, M., Velázquez, E., Willems, A. (2005). Reclassification of *Agrobacterium ferrugineum* LMG 128 as *Hoeflea marina* gen. nov., sp. nov. *Int J Syst Evol Microbiol*. 55(3), 1163–1166. https://doi.org/10.1099/ijs.0.63291-0.
76. Polish Collection of Microorganisms. https://pcm.org.pl/home (accessed 28 February 2025).
77. Regional Specialized Collection of Alkanotrophic Microorganisms. https://iegm.ru/iegmcol/index.html (accessed 28 February 2025).
78. Reports sent by Competent National Authorities to the European Commission. https://circabc.europa.eu/ui/group/3f466d71-92a7-49eb-9c63-6cb0fadf29dc/library/ddb37bea-f550-4504-ad31-3375dce1f37e (accessed 6 June 2023).
79. Resource Centre Culture Collection of Microorganisms. https://researchpark.spbu.ru/en/ccem-eng (accessed 28 February 2025).
80. Russian Collection of Agricultural Microorganisms. https://arriam.spb.ru/ (accessed 28 February 2025).
81. Russian National Collection of Industrial Microorganisms. https://vkm.ru/collecti.htm (accessed 28 February 2025).
82. Satomi, M., Vogel, B.F., Venkateswaran, K., Gram, L. (2007). Description of *Shewanella glacialipiscicola* sp. nov. and *Shewanella algidipiscicola* sp. nov., isolated from marine fish of the Danish Baltic Sea, and proposal that *Shewanella affinis* is a later heterotypic synonym of *Shewanella colwelliana. Int J Syst Evol Microbiol.* 57(2), 347–352. https://doi.org/10.1099/ijs.0.64708-0.
83. Schlesner, H., Rensmann, C., Tindall, B.J., Gade, D., Rabus, R., Pfeiffer, S., et al. (2004). Taxonomic heterogeneity within the *Planctomycetales* as derived by DNA-DNA hybridization, description of *Rhodopirellula baltica* gen. nov., sp. nov., transfer of *Pirellula marina* to the genus *Blastopirellula* gen. nov. as *Blastopirellula marina* comb. nov. and emended description of the genus *Pirellula*. *Int J Syst Evol Microbiol*. 54, 1567–1580. https://doi.org/10.1099/ijs.0.63113-0.
84. Senčilo, A., Luhtanen, A.-M., Saarijärvi, M., Bamford, D.H., Roine, E. (2015). Cold-active bacteriophages from the Baltic Sea ice have diverse genomes and virus-host interactions: comparative genomics of the sea-ice phages. *Environ Microbiol*. 17(10), 3628–3641. https://doi.org/10.1111/1462- 2920.12611.
85. Simris Biologics website. https://simrisbiologics.com (accessed 28 February 2025).
86. Spila website. https://spila.lt (accessed 28 February 2025).
87. Spirulina Nord website. https://spirulinanord.eu (accessed 28 February 2025).
88. Stante, M., Weiland-Bräuer, N., Repnik, U., Werner, A., Bramkamp, M., Chibani, C.M., et al. (2024). Four novel *Caudoviricetes* bacteriophages isolated from Baltic Sea water iInfect colonizers of *Aurelia aurita*. *Viruses*. 15(7), 1525. https://doi.org/10.3390/v15071525.
89. Strain Collection of Microalgae and Cyanobacteria from Northern and Arctic Regions in The Institute of Biology of Komi Scientific Centre. https://ib.komisc.ru/sykoa/eng/collection (accessed 28 February 2025).
90. Šulčius, S., Holmfeldt, K. (2016). Viruses of microorganisms in the Baltic Sea: current state of research and perspectives. *Mar Biol Res*. 12(2), 115–124. https://doi.org/10.1080/17451000.2015.1118514.
91. Šulčius, S., Šimoliūnas, E., Alzbutas, G., Gasiūnas, G., Jauniškis, V., Kuznecova, J., et al. (2019). Genomic characterization of cyanophage vB_AphaS-CL131 infecting filamentous diazotrophic cyanobacterium *Aphanizomenon flos-aquae* reveals novel insights into virus-bacterium interactions. *Appl Environ Microbiol.* 85(1), e01311-18. https://doi.org/10.1128/AEM.01311-18.
92. Šulčius, S., Šimoliūnas, E., Staniulis, J., Koreivienė, J., Baltrušis, P., Meškys, R., et al. (2015). Characterization of a lytic cyanophage that infects the bloom-forming cyanobacterium *Aphanizomenon flos-aquae*. *FEMS Microbiol Ecol.* 91(2), 1–7. https://doi.org/10.1093/femsec/fiu012.
93. Surup, F., Wieg, S., Boedeker, C., Heuer, A., Peeters, S.H., Jogler, M., et al. (2020). *Stieleria varia* sp. nov., isolated from wood particles in the Baltic Sea, constitutes a novel species in the family Pirellulaceae within the phylum *Stieleria varia* sp. nov., isolated from wood particles in the Baltic Sea, constitutes a novel species in the family Pirellulaceae within the phylum Planctomycetes. *Antonie Van Leeuwenhoek* 113(12), 1953–1963. https://doi.org/10.1007/s10482-020-01456-9.
94. Sustainable Exploitation of Bio-based Compounds Revealed and Engineered from Natural Sources Project website. https://cordis.europa.eu/project/id/101000794 (accessed 28 February 2025).
95. Szczecin Collection of Diatoms. https://www.facebook.com/marinebenthicdiatomproject/.
96. Szczecin Diatomological Collection. https://herbariumpomeranicum.pl.
97. Tartu Fungal Culture Collection. https://kogud.emu.ee (accessed 28 February 2025).
98. Teikari, J.E., Hou, S., Wahlsten, M., Hess, W.R., Sivonen, K. (2018). Comparative genomics of the Baltic Sea toxic cyanobacteria *Nodularia spumigena* UHCC 0039 and its response to varying salinity. *Front Microbiol.* 9, 356. https://doi.org/10.3389/fmicb.2018.00356.
99. The Algal Bank. https://gu.se/en/marina-vetenskaper/about-us/algal-bank-gumacc (accessed 28 February 2025).
100. The Bacterial Diversity Database BacDive https://bacdive.dsmz.de/ (accessed 28 February 2025).
101. The Collection of Cyanoprokaryotes of The Polar-Alpine Botanic Garden-Institute. https://isling.org/cyano (accessed 28 February 2025).
102. The International Escherichia and Klebsiella Centre (WHO). https://en.ssi.dk/surveillance-and-preparedness/national-reference-laboratories/escherichia-shigella-og-klebsiella (accessed 28 February 2025).
103. The Norwegian Culture Collection of Algae, NORCCA. https://norcca.scrol.net (accessed 28 February 2025).
104. Uchino, Y., Hamada, T., Yokota, A. (2002). Proposal of *Pseudorhodobacter ferrugineus* gen nov, comb nov, for a non-photosynthetic marine bacterium, *Agrobacterium ferrugineum*, related to the genus Rhodobacter. *J Gen Appl Microbiol.* 48(6), 309–319. https://doi.org/10.2323/jgam.48.309.
105. Uchino, Y., Hirata, A., Yokota, A., Sugiyama, J. (1998). Reclassification of marine *Agrobacterium* species: Proposals of *Stappia stellulata* gen. nov., comb. nov., *Stappia aggregata* sp. nov., nom. rev., *Ruegeria atlantica* gen. nov., comb. nov., *Ruegeria gelatinovora* comb. nov., *Ruegeria algicola* comb. nov., and *Ahrensia kieliense* gen. nov., sp. nov., nom. rev. *J Gen Appl Microbiol.* 44, 201–210. https://doi.org/10.2323/jgam.44.201.
106. Urzi, C., Salamone, P., Schumann, P., Rohde, M., Stackebrandt, E. (2004). *Blastococcus saxobsidens* sp. nov., and emended descriptions of the genus *Blastococcus* Ahrens and Moll 1970 and *Blastococcus aggregatus* Ahrens and Moll 1970. *Int J Syst Evol Microbiol.* 54, 253–259. https://doi.org/10.1099/ijs.0.02745-0.
107. Vandieken, V., Marshall, I.P.G., Niemann, H., Engelen, B., Cypionka, H. (2018). *Labilibaculum manganireducens* gen. nov., sp. nov. and *Labilibaculum filiforme* sp. nov., novel Bacteroidetes isolated from subsurface sediments of the Baltic Sea. *Front Microbiol*. 8, 2614. https://doi.org/10.3389/fmicb.2017.02614.
108. Vandieken, V., Niemann, H., Engelen, B., Cypionka, H. (2017). *Marinisporobacter balticus* gen. nov., sp. nov., *Desulfosporosinus nitroreducens* sp. nov. and *Desulfosporosinus fructosivorans* sp. nov., new spore-forming bacteria isolated from subsurface sediments of the Baltic Sea. *Int J Syst Evol Microbiol* 67(6), 1887–1893. https://doi.org/10.1099/ijsem.0.001883.
109. Voss, B., Bolhuis, H., Fewer, D.P., Kopf, M., Möke, F., Haas, F., et al. (2013). Insights into the physiology and ecology of the brackish-water-adapted cyanobacterium *Nodularia spumigena* CCY9414 based on a genome-transcriptome analysis. *PLoS One.* 8(3), e60224. https://doi.org/10.1371/journal.pone.0060224.
110. VTT Technical Research Centre of Finland Ltd. https://culturecollection.vtt.fi/ (accessed 28 February 2025).
111. Weihenstephan Microbial Strain Collection. https://iel.tum.de/ziel/ueber-uns/alumni/ziel-abteilung-mikrobiologie-lehrstuhl-fuer-mikrobielle-oekologie/microbial-strain-collection/ (accessed 28 February 2025).
112. WFCC Estonian Human Microbiota Biobank. https://https://eemb.ut.ee/humb_eesti_Contacts_list.php (accessed 28 February 2025).
113. Wiegand, S., Jogler, M., Boedeker, C., Pinto, D., Vollmers, J., Rivas-Marín, E., (2020). Cultivation and functional characterization of 79 planctomycetes uncovers their unique biology. *Nat Microbiol*. 5(1), 126–140. https://doi.org/10.1038/s41564-019-0588-1.
114. Xu, L., Sun, C., Fang, C., Oren, A., Xu, X.W. (2020). Genomic-based taxonomic classification of the family Erythrobacteraceae. *Int J Syst Evol Microbiol.* 70(8), 4470–4495. https://doi.org/10.1099/ijsem.0.004293.
115. Yeast Collection of the Soil Biology Department in Lomonosov Moscow State University (KBP MSU or KBP). https://depo.msu.ru (accessed 28 February 2025).
116. Ziemke, F., Höfle, M.G., Lalucat, J., Rosselló-Mora, R. (1998). Reclassification of *Shewanella putrefaciens* Owen's genomic group II as *Shewanella baltica* sp. nov. *Int J Syst Bacteriol*. 48, 179–186. https://doi.org/10.1099/00207713-48-1-179.
